# Advancements in nanomaterials for nanosensors: a comprehensive review

**DOI:** 10.1039/d4na00214h

**Published:** 2024-05-24

**Authors:** Moustafa A. Darwish, Walaa Abd-Elaziem, Ammar Elsheikh, Abdelhameed A. Zayed

**Affiliations:** a Physics Department, Faculty of Science, Tanta University Tanta 31527 Egypt mostafa_ph@yahoo.com mostafa_ph@science.tanta.edu.eg; b Department of Mechanical Design and Production Engineering, Faculty of Engineering, Zagazig University P.O. Box 44519 Egypt; c Department of Materials Science and Engineering, Northwestern University Evanston IL 60208 USA; d Production Engineering and Mechanical Design Department, Faculty of Engineering, Tanta University Tanta 31521 Egypt; e Department of Industrial and Mechanical Engineering, Lebanese American University P.O. Box 36 / S-12 Byblos Lebanon

## Abstract

Nanomaterials (NMs) exhibit unique properties that render them highly suitable for developing sensitive and selective nanosensors across various domains. This review aims to provide a comprehensive overview of nanomaterial-based nanosensors, highlighting their applications and the classification of frequently employed NMs to enhance sensitivity and selectivity. The review introduces various classifications of NMs commonly used in nanosensors, such as carbon-based NMs, metal-based NMs, and others, elucidating their exceptional properties, including high thermal and electrical conductivity, large surface area-to-volume ratio and good biocompatibility. A thorough examination of literature sources was conducted to gather information on NMs-based nanosensors' characteristics, properties, and fabrication methods and their application in diverse sectors such as healthcare, environmental monitoring, industrial processes, and security. Additionally, advanced applications incorporating machine learning techniques were analyzed to enhance the sensor's performance. This review advances the understanding and development of nanosensor technologies by providing insights into fabrication techniques, characterization methods, applications, and future outlook. Key challenges such as robustness, biocompatibility, and scalable manufacturing are also discussed, offering avenues for future research and development in this field.

## Abstract 2

Nanomaterials (NMs) exhibit unique properties that render them highly suitable for developing sensitive and selective nanosensors across various domains.

## Graphical abstract

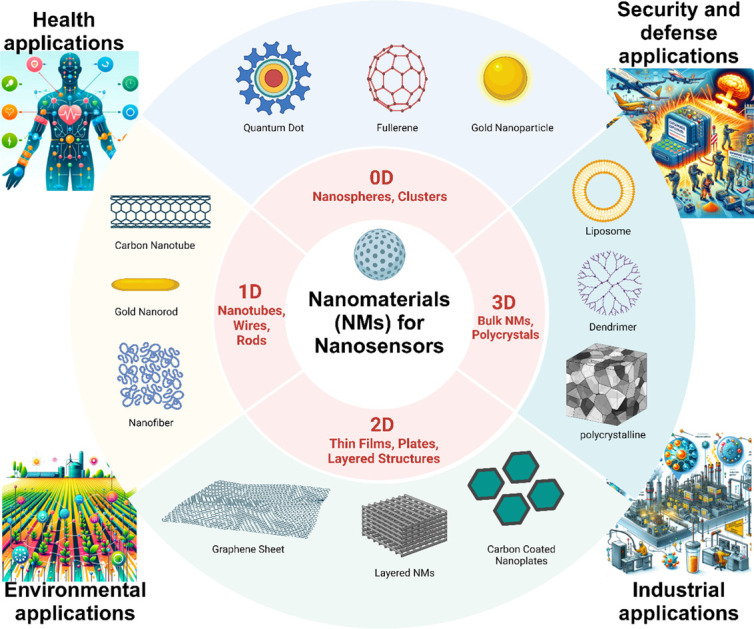

## 1. Introduction

In recent years, the rapid evolution of nanotechnology has led to groundbreaking developments in nanosensors, revolutionizing their design, fabrication, and applications. Nanosensors, characterized by their miniature dimensions and exceptional sensitivity, have emerged as pivotal tools in various fields, from healthcare and environmental monitoring to industrial processes and security applications.^1–3^ At the heart of these nanosensors lie innovative NMs, the building blocks that dictate their performance, selectivity, and responsiveness. Significant research efforts over the past few decades have focused on developing novel NMs to serve as recognition elements in nanosensors and transduce signals with extremely high fidelity and sensitivity.^4–6^ NMs provide unique size-dependent properties, large surface-to-volume ratios, tunability of physical properties, and surface chemistry, which make them ideal for interfacing with target analytes. Many NMs, spanning carbon nanotubes, graphene, metal and metal oxide nanoparticles (NPs), quantum dots, and nanowires, have been explored for fabricating the next generation of nanosensors.^7,8^

Considerable research has focused on enhancing the intrinsic properties of these NMs, formulating stable surface chemistries, and their integration within sensing devices to harness their potential fully. This has led to exponential growth in the variety of highly efficient and robust nanomaterial-enabled optical, electrochemical, and mass-sensitive detection platforms for sensing applications.^9,10^ Nanomaterial-based transduction mechanisms in these sensors include changes in conductance, resonant frequency, absorption/fluorescence, *etc.*, upon interaction with target analytes. Innovations in nanofabrication have enabled precise control over the dimensions of NMs down to a few nanometers, drastically improving sensor performance metrics like sensitivity and lower detection limits.

The discovery of novel low-dimensional NMs like 2D transition metal dichalcogenides with thickness-dependent bandgaps has opened new avenues for their application in susceptible photoluminescence-based sensing schemes.^11,12^ Further, recent research has focused on the heterogeneous integration of multiple NMs, enabling synergistic combinations of desirable attributes from individual components to enhance sensor parameters multifold over their single-component counterparts.

This review thoroughly investigates the synergy between NMs and nanosensors, shedding light on their collaborative potential across various applications. It delves into the specific role of NMs in shaping nanosensors, covering diverse types such as carbon-based, metal-based, and semiconductor-based NMs. Applications range from healthcare and environmental monitoring to the industrial and defense sectors. The review also touches on integrating nanosensors with artificial intelligence, offering insights into future possibilities. Further, along with prospects, key challenges related to nanomaterial synthesis, device fabrication workflows, and improving robustness for practical implementation are discussed. By addressing both advantages and challenges, this review provides a comprehensive understanding of the current landscape and anticipates potential developments in nanomaterial-based nanosensors.

## 2. History of nanotechnology

The origins of nanotechnology can be traced back to the early stages of human civilization, with evidence of the first utilization of NMs dating back to the ninth century in Mesopotamia. Artisans from this period unknowingly employed NMs to create a sparkling effect on pottery surfaces. The luminous quality of the ceramic surfaces resulted from the homogeneous dispersion of silver and copper NPs within the glassy matrix.^13^ During these ancient times, artisans did not use the term “nanomaterials”.^14^ Ancient artifacts demonstrate various instances of employing nanocomposites. Examples include the Lycurgus cup (400 AD) with gold-silver alloy NPs to alter glass color,^15^ durable Damascus steel swords (300–1700 AD) forged with integrated NPs,^16^ and Maya Blue pigment (800 AD), an enduring blue pigment composed of dye-absorbing clay with nanopores.^17,18^ However, the field emerged in the late 1900s, following visionary conceptual foundations and pioneering microscopy advances enabling direct observation and manipulation at the nanoscale.

In the early 1960s, Stephen Papell *et al.*^19^ developed magnetic colloidal suspensions using magnetite NPs dispersed in paraffin with oleic acid to prevent agglomeration. Subsequently, similar suspensions were prepared with various pure metal NPs in different carrier fluids.^20^

NPs have been found within many organisms for biological functions: bacteria, algae, insects, birds, and mammals. In 1975, Blakemore discovered biogenic magnetite NPs (Fe_3_O_4_), which humans use for magnetic orientation and navigation. In recent years, numerous advancements have been witnessed in the evolution of nanotechnology. In the 1980s, advanced microscopy techniques, such as scanning tunneling and atomic force microscopy, marked a breakthrough, enabling nanostructure's direct imaging and manipulation. This instrumental progress allowed for observation and control at the nanoscale. In 1985, the discovery of buckyballs, a spherical fullerene carbon allotrope, introduced a new class of NMs, sparking significant interest. The discovery of carbon nanotubes in 1991 propelled NMs to the forefront, advancing applications in electronics, materials, and biomedicine.

Pioneering nanosensor work in the early 2000s involved applications with carbon nanotubes, nanowires, and NPs for chemical and biosensing purposes. Important milestones catalyzing the field's evolution included developments like magnetic resonance force microscopy, achieving single electron spin detection by the early 1990s, micromechanical cantilever sensors by the late 1990s, and graphene demonstrating ultrasensitive biomolecule detection by 2004. These and other advances spawned proof-of-concept nanosensor demonstrations across optical, electrochemical, magnetic, and scanning probe modalities. Today, nanotechnology has evolved into broad multidisciplinary sensing fields, intersecting with biotechnology, artificial intelligence, electronics, materials, energy, medicine, and consumer goods, with ongoing expansion and advancements.

## 3. A brief overview of nanomaterials (NMs)

A brief overview of the definition, properties, types, and classifications of NMs is presented in this section. At the same time, the NMs frequently used in nanosensors are discussed in detail in later sections.

### 3.1. Definition and properties

NMs represent a fascinating and rapidly evolving field at the intersection of science and technology. They offer unique properties and applications due to their nanoscale dimensions. In a brief overview, NMs are defined as materials with structures or components at the nanometer scale, typically ranging from 1 to 100 nanometers.^21,22^ At this scale, materials often exhibit distinctive properties, including quantum effects and increased surface area, which can significantly differ from those observed in bulk materials.^23,24^ This is because materials have an extremely high surface area-to-volume ratio^25–27^ when structured or manipulated at the nanoscale. With a much greater proportion of atoms on material surfaces rather than the bulk interior, nanostructured variants display different optic, electronic, magnetic, mechanical, thermal, and chemical behaviors.

### 3.2. Types of nanomaterials (NMs)

Common types of NMs include nanoparticles (NPs), nanotubes, nanowires, nanocomposites, and quantum dots, each possessing unique characteristics that make them suitable for specific applications. NPs, for instance, are particles with dimensions at the nanoscale, while nanotubes are cylindrical structures with diameters at the nanoscale. NMs can be classified based on three main factors, *i.e.*, dimensionality, types, and morphology.

#### 3.2.1 Nanomaterials (NMs) classification by dimensionality

**Fig. 1:**
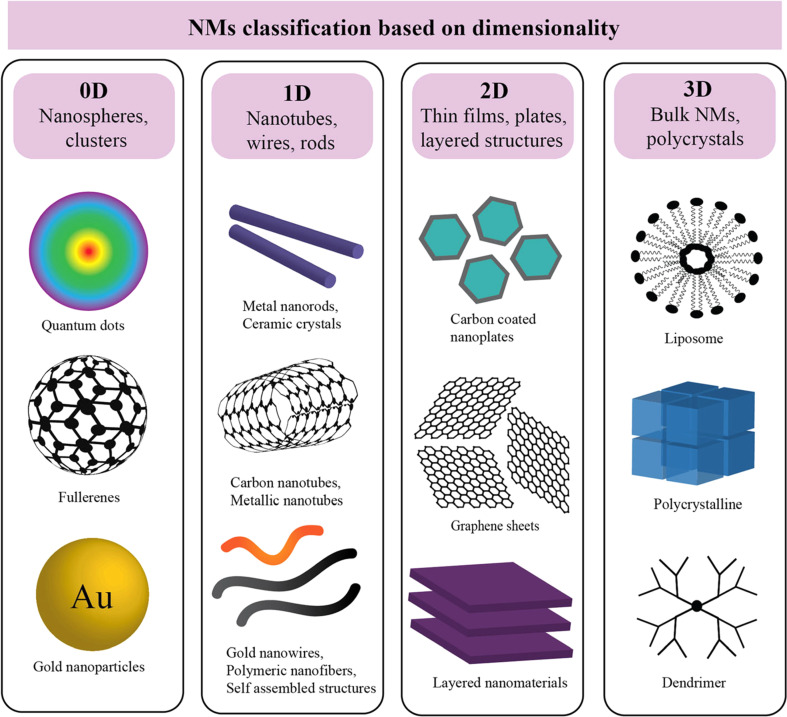
Schematic of nanoparticles by dimensionality with examples: 0D (nanoparticles), 1D (nanorods), 2D (films), and 3D nanocomposites, where synergistic properties emerge from distinct materials combined at the nanoscale.^57^

NMs can be classified based on their dimensionality, as presented in Fig. 1, considering the following categories.

##### 3.2.1.1. Zero-dimensional (0D) NMs

**Fig. 2:**
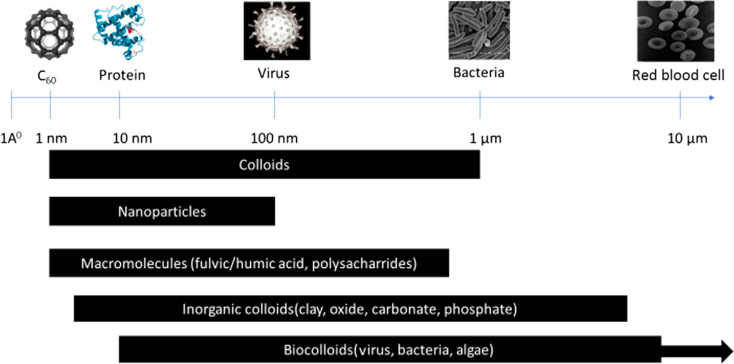
Size comparison of different structures (living and nonliving).^58^

Zero-dimensional NMs refer to nanostructures where all three external dimensions are confined to the nanoscale. This includes: (1) quantum dots (QDs): quantum dots (*e.g.*, CdSe, InP) are semiconductor nanocrystals typically ranging from 2 to 10 nanometers in diameter.^28–30^ Their size-dependent electronic properties make them valuable for applications in imaging, solar cells, and displays. (2) Fullerenes: fullerenes, such as C60, are closed-cage carbon molecules with a diameter of approximately 0.7 nanometers.^31^ These spherical structures exhibit unique electronic and chemical properties, contributing to applications in drug delivery, sensors, and materials science. (3) Nanoparticles (NPs): NPs (*e.g.*, gold and silver) and metal oxide NPs (*e.g.*, Fe_2_O_3_, TiO_2_), generally spherical, have dimensions in the nanoscale, typically between 1 and 100 nanometers. Fig. 2 illustrates a size comparison of NPs with various living and nonliving species. They encompass multiple materials and find applications in catalysis, medicine, and nanocomposite fabrication. (4) Metal clusters: metal clusters consist of a small number of metal atoms aggregated into a nanoscale structure. These clusters exhibit size-dependent electronic and catalytic properties, making them relevant for catalysis and electronic applications.

##### 3.2.1.2. One-dimensional (1D) NMs

One-dimensional (1D) NMs are characterized by having one dimension at the nanoscale. This category includes a variety of NMs with unique structures and applications: (1) nanowires: nanowires are elongated structures with diameters typically in the range of 1 to 100 nanometers and lengths extending from nanometers to micrometers.^32,33^ They find applications in electronics, sensors, and nanocomposite materials. (2) Nanotubes: nanotubes, such as carbon nanotubes (CNTs), are hollow cylindrical structures with diameters as small as 0.4 nanometers and lengths ranging from nanometers to millimeters.^34^ CNTs exhibit exceptional mechanical, electrical, and thermal properties, making them valuable in materials science, electronics, and nanocomposite development.^35–37^ (3) Nanorods: nanorods are rod-shaped structures with diameters typically ranging from 1 to 100 nanometers and lengths extending up to micrometers.^38,39^ They are employed in various applications, including catalysis, sensors, and biomedical devices.^40,41^ (4) Nanofibers: nanofibers are elongated structures with diameters in the nanoscale and varying lengths. They are widely used in tissue engineering, filtration, and electronic devices.^42,43^

##### 3.2.1.3. Two-dimensional (2D) NMs

Two-dimensional (2D) NMs are characterized by having dimensions in the nanoscale in two directions, forming thin layers or sheets. This category includes various materials with diverse properties and applications: (1) graphene: graphene is a single layer of carbon atoms arranged in a hexagonal lattice with a thickness of approximately 0.34 nanometers.^44^ It exhibits exceptional electrical conductivity, mechanical strength, and thermal conductivity, making it valuable in electronics, materials science, and energy storage.^45^ (2) Transition metal dichalcogenides (TMDs): TMDs, such as molybdenum disulfide (MoS_2_) and tungsten diselenide (WSe_2_), are layered materials with thicknesses in the nanoscale.^46^ They possess unique electronic and optical properties, contributing to applications in electronics, optoelectronics, and catalysis.^47^ (3) Hexagonal boron nitride (h-BN): h-BN is a two-dimensional material with a hexagonal lattice structure similar to graphene. It is an insulator with high thermal conductivity, finding applications in electronics, thermal management, and lubrication.^48,49^ (4) Black phosphorus (BP): BP is a layered material with a thickness varying in the nanoscale. It exhibits a tunable bandgap, making it suitable for applications in electronics, photodetectors, and energy storage.^50,51^

##### 3.2.1.4. Three-dimensional (3D) NMs

Three-dimensional (3D) NMs are characterized by nanostructures with nanoscale dimensions in all three directions, resulting in complex 3D architectures. This category includes various materials with distinct properties and applications: (1) nanocomposites: nanocomposites consist of NPs dispersed within a three-dimensional matrix. The size of the NPs and their distribution within the matrix are critical factors influencing the properties of the nanocomposite material.^52,53^ Applications include lightweight structural materials and advanced coatings. (2) Nanoarchitectures: nanoarchitectures involve the deliberate design and construction of three-dimensional nanoscale structures with precise control over their dimensions and configurations. These structures find applications in drug delivery, catalysis, and energy storage.^54^ (3) 3D nanoporous materials: nanoscale pores are distributed in three dimensions, offering high surface area and unique adsorption properties. Applications include gas storage, separation, and catalysis. (4) 3D nanowire networks: Networks formed by interconnected nanowires in three dimensions, providing enhanced electrical and mechanical properties.^55,56^ These networks are utilized in electronics, sensors, and energy devices.

#### 3.2.2 Nanomaterials (NMs) classification by type

**Fig. 3:**
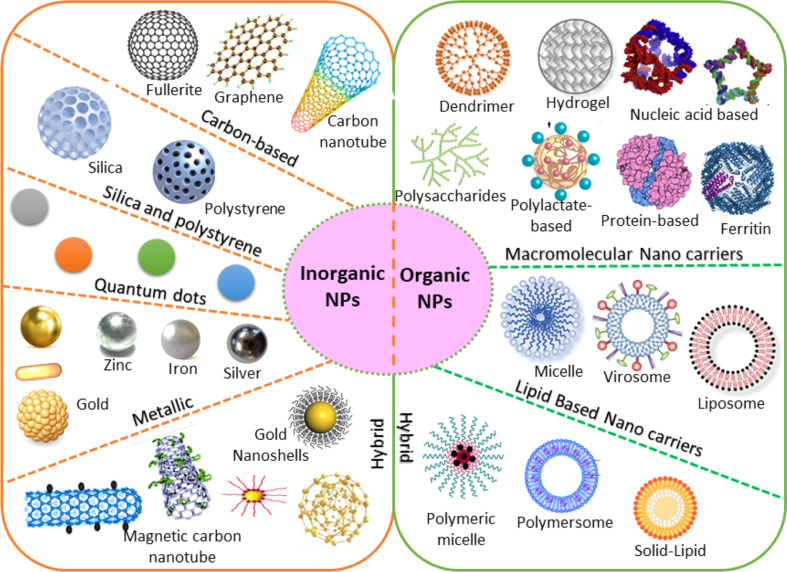
Various configurations of organic and inorganic nanomaterials.^60^

NMs can be systematically classified into four main categories based on their composition, which involve: (i) carbon-based NMs: these materials constitute a diverse class characterized by carbon atoms in various morphologies and phases, as discussed later. Fullerenes like C60 exhibit ellipsoidal or spherical structures, carbon nanotubes (CNTs) manifest as hollow wire-like tubes, and carbon nanofibers form nanowires. Additionally, carbon black exists in particulate form, and graphene consists of layers of exfoliated graphite. Carbon onions, another allotrope, also fall within this category. These materials are produced through various methods, such as laser ablation, arc discharge, and chemical vapor deposition (CVD). The versatility of carbon-based NMs makes them pivotal in numerous applications, from electronics to medicine. (ii) Inorganic-based NMs: inorganic-based NMs comprise particles made from metals or metal oxides, each measuring less than 100 nanometers. This classification encompasses pure precious metal NPs like gold (Au) or silver (Ag), metallic oxides such as titanium dioxide (TiO_2_) and zinc oxide (ZnO), as well as semiconductors like silicon and various ceramics,^21,59^ as depicted in Fig. 3. These NMs exhibit unique properties and find applications in a broad spectrum of fields, including catalysis, electronics, and biomedical research. (iii) Organic-based NMs: organic-derived NMs encompass NMs and nanostructures comprised of organic substances. Dendrimers, micelles, liposomes, and polymers can be generated by applying non-covalent (weaker) interactions, capitalizing on self-assembly properties, or intentional molecular design. (iv) Composite-based NMs: composite-based NMs refer to nanostructures of different materials. These materials are intricate, multiphase nanostructures featuring at least one phase on the nanoscale. These materials can amalgamate NPs of diverse compositions and shapes or incorporate NPs within bulk-type materials, such as hybrid nanofibers or intricate structures like metal–organic frameworks. The nanocomposites encompass mixtures of carbon-based, metal-based, or organic-based NMs with various forms of bulk materials, including metals, ceramics, and polymers. Composite-based NMs find applications in multiple fields, including electronics, materials science, and medicine, where their tailored properties contribute to enhanced performance and functionality.

#### 3.2.3 Nanomaterials (NMs) classification by morphology

**Fig. 4:**
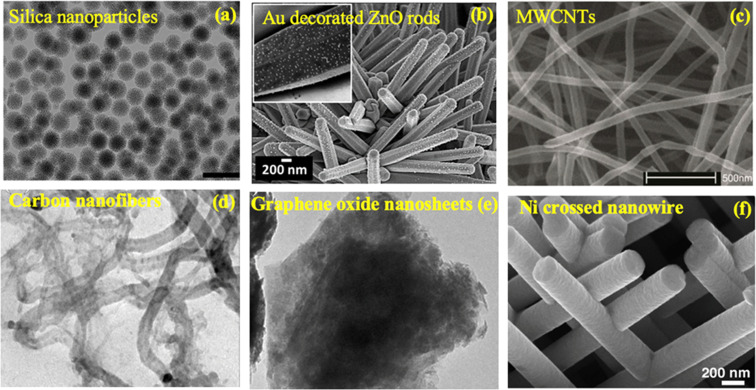
Nanomaterials with different morphologies: (a) silica nanoparticles^62^, (b) Au decorated ZnO rods^63^, (c) MWCNTs^64^, (d) 1D carbon nanofibers^65^, (e) graphene oxide nanosheets^65^, and (f) Ni crossed nanowire.^66^

NMs exhibit a wide range of morphologies, providing a basis for their classification. The dimensions and shapes of NMs play a crucial role in determining their properties and applications. One classification criterion involves the structural arrangement, distinguishing between aggregate and agglomerate structures, which are influenced by the interactions at the nanoscale. Beyond this, NMs can be categorized based on their specific shapes and spatial organizations. Examples include NPs with diverse shapes, such as spheres, rods, tubes, needles, cubes, and octahedrons, as shown in Fig. 4, each achievable through variations in synthesis methods and conditions. Additionally, high-aspect-ratio particles like nanotubes and nanowires stand in contrast to low-aspect-ratio NPs with shapes like spheres and cubes.^61^ The morphological diversity extends to organic NMs, offering complex architectures through self-assembling methods, further highlighting the importance of morphology in tailoring nanomaterial properties for specific applications.

## 4. Nanomaterial (NMs) used in nanosensors

**Fig. 5:**
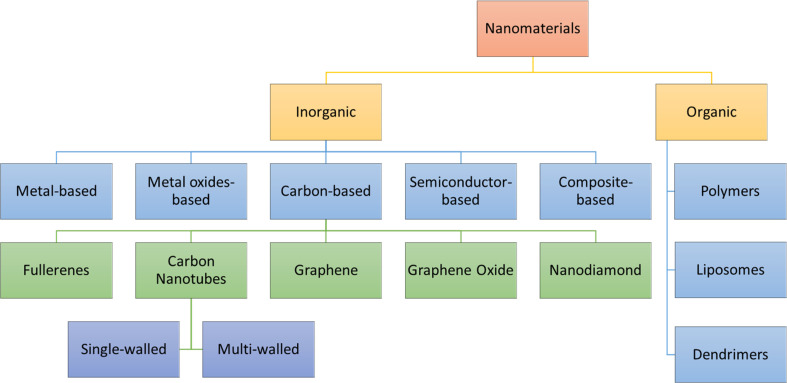
Classifications of nanomaterials based on chemical nature.^68^

The present section discusses the NMs that have been extensively used in nanosensors. Based on their chemical nature, NMs can be classified, as shown in Fig. 5. The two main categories are inorganic and organic NMs.^67^ The organic ones are NMs synthesized from proteins, phospholipids, polymers, and hybrids. This group of NMs includes polymers, dendrimers, and liposomes. On the other hand, metals and alloys, metal oxides, semiconductor oxides, composites, and carbon structures are classified as organic. Also, the motivation to create intricate devices encompassing nanoscale materials, biological components, and advanced materials, collectively known as nano biosensors, stems from the growing need to detect a diverse array of molecules at low concentrations with high specificity. For the design and fabrication of nanosensors based on NMs, the key is the creation of sophisticated NMs with regulated functionality, directed size, shape, nature, and crystallinity. The frequently used NMs for this purpose are introduced in the following subsection, which discusses their structures and properties.

### 4.1. Carbon-based nanomaterials (NMs)

The remarkable features and impressive applications of carbon-based nanomaterials (CBNs), including carbon nanotubes, graphene, fullerenes, and carbon fibers, have made them extremely popular. These CBNs are highly flexible, highly conductive, highly mechanically strong, and have great electron mobility with anisotropic heat conductivity.^69,70^ Moreover, CBNs introduce excellent stability and minimal toxicity that are environmentally friendly due to their construction of pure carbon.^71^ Because of their extraordinarily high mechanical, thermal, optical, electrical, and magnetic capabilities, these CBNs are regarded as key components of advanced nanotechnology and smart NMs. These could be used in several industries, including biomedical applications, solar cells, sensors, optoelectronics, medication delivery, environmental monitoring, energy storage devices, and catalysis.

#### 4.1.1 Fullerenes

The widely-known allotropes of carbon, known for their distinct properties, include amorphous carbon, diamond, and graphite. The allotropic form strongly affects the physical properties of carbon. Buckminsterfullerene's, or simply fullerene, discovered by Kroto *et al.* 1985,^72^ is the first form of CBNs. Also, they proved the stability of this new material in the gaseous state.

Within the fullerene family, C60 stands out as the most significant member. Comprising 20 hexagons and 12 pentagons, it boasts 32 surfaces and 60 vertices. Resembling a soccer ball, its iconic spherical shape has earned it the renowned name “buckyball,” depicted schematically in Fig. 1.^73^ Also, there are other fullerene structures, such as C70, C76, and C78, which are larger than C60, while C28 and C36 have relatively smaller structures. Fullerene is classified as 0D material, which has a face-centered cubic (FCC) lattice structure, and its type of crystal planes are 111 and 200 at room temperature.^74^ At elevated temperatures above 677 °C, the FCC is transformed into a graphite-like arrangement.

#### 4.1.2 Carbon nanotubes

Sumio Iijima, in 1991,^75^ discovered a new carbon-based nanomaterial called carbon nanotubes (CNTs). These CNTs can be considered a form of fullerenes. The first observation of Iijima during carbon arc discharge was multi-wall nanotubes (MWNT), followed by the observation of single-walled carbon nanotubes (SWNT)^76^ after two years in 1993. This tube-like structure material of graphite has been used in diversified applications, especially nanosensors, due to its exceptional physical, structural, and chemical properties. These properties account for the nanotube's wall nature, length, diameter, and type. The tubular shape is formed by rolling graphene sheets. Although CNTs have diameters of small nanometers, their length can be several millimeters, and they are considered 1D materials with aspect ratios higher than 333 and high surface-to-volume ratios. The external diameters of the MWNT range from 0.4 to 2 nm,^77^ while the outer diameter range of the SWNT is from 2 to 100 nm.^78^

Additionally, SWNT can exhibit metallic or semiconducting behavior, depending on the diameter and chiral angle.^79^ CNTs can carry high current without high heating effects because they possess an anisotropic dielectric property.^75^ CNTs are preferred for sensing the application of gases with low energy consumption due to their high efficiency in detecting gases at room temperature without the need for elevated temperatures. Catalyzed chemical vapor deposition (CVD),^80^ laser method,^81^ and arc plasma^82^ are the most widely used methods for CNTs synthesis. The insolubility, as well as the tendency to agglomerate, are the main disadvantages of CNTs.

#### 4.1.3 Graphene

Another carbon allotrope is graphene, which consists of a single layer of atoms in a hexagonal lattice arrangement,^83^ showing durability 200 times greater than steel and 40 times greater than diamond,^84^ making it the strongest, lightest, and toughest material ever discovered. Besides graphene's extraordinary electrical, optical, mechanical, and thermal properties, excellent electrical and thermal conductivity and noticeable light absorption are the most critical characteristics.^26,85^ Graphene was discovered in 2004 by A. K. Geim and Novoselov^86^ and is classified as a 2D material with the thinnest structure and very high surface area. Graphene consists of sp^2^ carbon-bonded atoms with a honeycomb lattice.^87^ Multiple sheets can be stacked together to form a multilayer form, resulting in a 3D structure.

Moreover, graphene is the basic building block for other CBNs as it can be wrapped up into 0D fullerenes, rolled into 1D nanotubes, or stacked into 3D graphite. Initially isolated through mechanical exfoliation using the method of “scotch tape,” graphene can be prepared using various techniques. Sonicating the graphite intercalation compounds in surfactants or stabilizers is a form of the “top-down” approach for graphene production.^88,89^ Also, graphite and its derivatives can be utilized to produce graphene by liquid phase exfoliation^90,91^ or electrochemical exfoliation in the case of graphite anode with the aid of ionic liquid.^92,93^ In addition, graphene can be produced from hydrocarbons by the chemical vapor deposition process.^94^ At the same time, epitaxial growth is employed for the production of graphene on metal surfaces^95,96^ along with the sublimation of silicon carbide (SiC) in ultrahigh vacuum to produce the hexagonal lattice arrangement of carbon on the SiC substrate after the desorption of Si.^97,98^ An essential feature of graphene and CNTs that has been exploited in numerous types of nanosensors, especially pollution detection sensors, is the easy transfer of electrons between electroactive species and electrodes.

#### 4.1.4 Graphene oxide

Graphene oxide (GO) can be produced by adding oxygen-containing groups to the surface of graphite^99^ and other electrochemical oxidation.^100^ Graphene oxide, a 2D material formed by sp^3^ bonding between oxygenated functionalities and carbon atoms, is employed as an insulating substance.^101^ Graphene oxide, whether single or multilayer, demonstrates favorable electrical conductance, expansive specific surface area, elevated electrochemical reactivity, and compatibility with living organisms. These attributes are indispensable in effectively advancing sensing devices.^102^ Although GO is chemically more active than graphene, it shows less electrical conductivity, aside from the disability of visible light absorption.^103^ The oxidation of graphite was performed using different methods, such as Staudenmaier's, Hofmann's, and Hummers' methods.^104^ Still, these methods have the disadvantages of emitting toxic gases and producing a mixture of GO/graphite due to the imperfect oxidation process. Using Hummers's method, a 0.80 nm inter-planar distance GO can be generated from graphite. The process is conducted in two steps: initially, NaNO_3_, H_2_SO_4_, H_3_PO_4_, and KMnO_4_ are added to create graphite oxide from graphite powder. Subsequently, graphene oxide (GO) is obtained by sonicating the graphite oxide.^105^ Recently, multiple endeavors have been conducted to achieve graphene oxide with a substantial level of oxidation using diverse adaptations made to the Hummers' synthesis method.^99,106^

#### 4.1.5 Nanodiamonds

**Fig. 6:**
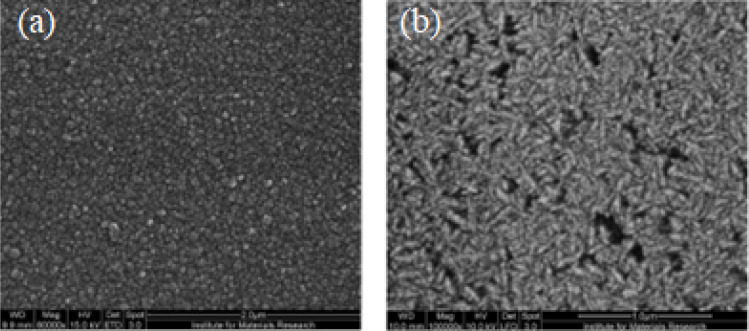
Images of (a) nanocrystalline and (b) ultra-nanocrystalline diamond using the scanning electron micrograph.^107^

Nanodiamond, or nanocrystalline diamond, encompasses a wide range of materials. It is commonly acknowledged that nanocrystalline diamond (NCD) consists of facets with dimensions smaller than 100 nm. Conversely, the term “ultra-nanocrystalline diamond” (UNCD) has been coined to describe materials with grain sizes less than 10 nm. The disparities in morphology arise from the growth process, and the structure images of nanocrystalline and ultra-nanocrystalline diamonds using the scanning electron micrograph are shown in Fig. 6.^107^

Nanodiamonds possess exceptional mechanical and optical properties, substantial surface areas, and adjustable surface structures. Additionally, they are non-toxic, rendering them highly suitable for biomedical applications, particularly biosensors.^108,109^ These nanodiamonds have been synthesized through various methods, including the detonation technique,^110^ plasma-enhanced chemical vapor deposition (CVD),^111^ graphite ion irradiation,^112^ high-energy ball milling of high-pressure high-temperature (HPHT) diamond microcrystals,^113^ and laser ablation.^114^

#### 4.1.6 Other carbon-based nanomaterials (CBNs)

In addition to the previously mentioned CBNs, other NMs have been introduced for use in nanosensors, particularly electrochemical ones. Such materials include carbon nanofibers (CNFs), carbon nanospheres, and mesoporous carbon. These materials have extinct features such as one-direction accelerated electron transfer, essential for nanosensors with a lower detection time.^115,116^ Electrospinning is considered a suitable method for producing different porosity percentages and diameter interconnected CNFs despite the difficulty of the process conditions control.^117^ To improve the sensing behavior of the CNFs, other studies investigated the addition of various NPs to decorate the CNFs.^118–120^ Because of their high electrical conductivity, as well as their high adsorption ability and improved surface area, carbon mesoporous materials have been widely used in biosensors.^121,122^ Developing a removable template is an excellent challenge in producing mesoporous carbon due to the remarkable decrease in sensor sensitivity caused by the incomplete removal of such template materials. Polymer 3D structures and silicon are used as template materials to partially overcome this limitation.

**Fig. 7:**
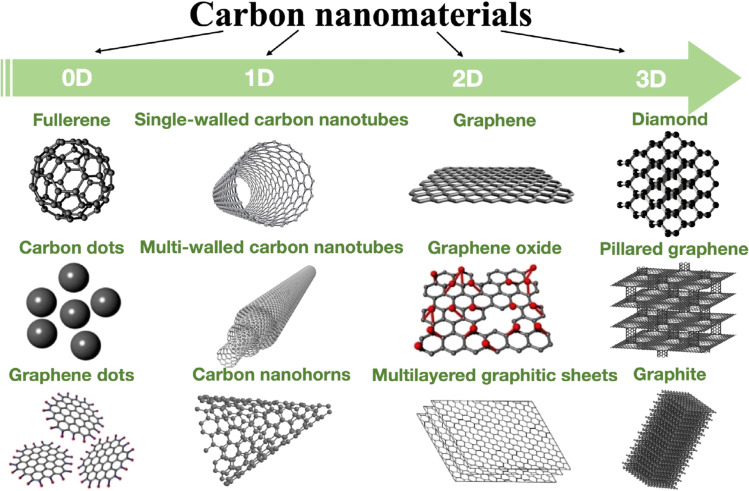
Dimensionality of various allotropes of carbon.^130^

Many methods, including X-ray photoelectron spectroscopy (XPS),^123,124^ laser scanning microscopy,^125^ Raman spectroscopy,^126,127^ energy dispersive spectroscopy (EDS), transmission electron microscopy (TEM),^128^ scanning electron microscopy (SEM),^129^ atomic force microscopy (AFM), optical microscopy, as well as conductivity measurements and electrochemistry, have been used for characterizing CBNs. Fig. 7 shows the dimensionality of some carbon allotropes.

### 4.2. Metal-based nanomaterials (NMs)

The ability to generate metal NPs within the range of 1 to 100 nm has resulted in numerous applications in nanosensor research. These NPs possess distinct characteristics, such as a high surface concentration of atoms/molecules and a significant surface-to-volume ratio. These can be harnessed to enhance detection and sensing in food-packing industries and healthcare-related disciplines. Metal NPs can be utilized independently or in conjunction with other nanostructures, amplifying signals, increasing sensitivity, and achieving substantial advancements in the nanosensors industry. The involvement of metallic NPs in biological and chemical sensing is closely tied to their physicochemical properties.^131^

Noble metals characterized by their high oxidation and corrosion resistance at high temperatures, namely platinum (Pt), silver (Ag), gold (Au), palladium (Pd), and ruthenium (Ru) NPs, have gained significant popularity and have been subjected to extensive research. Although these noble metals exhibit chemical inactivity in their macroscopic state, they exhibit distinctive physiochemical characteristics at the nanoscale.^132^

The categorization of noble metals NPs synthesis methodologies comprises two main approaches: “bottom-up” and “top-down”. Physical manipulation techniques such as pyrolysis, lithography, and micro-patterning are the core of the top-down approaches. At the same time, the bottom-up methods employ chemical transformations, including microwave synthesis, microemulsion techniques, and chemical reduction.^133^ Each method has its advantages and disadvantages. Top-down methods permit large-scale production without the need for chemical purification. However, the resulting NPs exhibit a wider range of sizes and varying morphologies, besides the difficulty of controlling synthesis parameters.

Conversely, a specific morphology and size, as well as easy control of the parameters, can be achieved by bottom-up methods. Still, it is not easy to attain large-scale production. Also, the bottom-up approaches are cheaper than the top-down ones.^134^

#### 4.2.1 Gold nanoparticles (NPs)

Gold NPs can be conveniently produced in sizes ranging from 3 to 100 nm in diameter, exhibiting diverse shapes. The synthesis of gold NPs through chemical reduction encompasses several methodologies, such as the seeding-growth method, the Turkevich method, green synthesis, the ascorbic acid synthesis, the NaBH_4_ synthesis with or without citrate, and the utilization of other reducing agent methods.^135^ It is widely acknowledged that the size, shape, and functionality of gold NPs are significantly affected by the chemical and physical parameters employed during their synthesis, including the reaction temperature, stirring rate, and the gold/reducing agent ratio. Gold NPs have many applications in nanosensor technology due to numerous advantages encompassing minimal toxicity, magnificent biocompatibility, and a high surface-to-volume ratio. In particular, gold NPs are used in optical nanosensors and biosensors, which utilize biological molecules such as carbohydrates, antibodies, nucleic acids, and enzymes to identify the desired biological phenomenon.^136^ Green synthesis using biological methods is employed as an environmentally benign and economical method to overcome the limitations of chemical and physical approaches, such as flammable substances and toxic chemicals. Amino acids, carboxylic acids, ketones, amines, enzymes, flavonoids, proteins, alkaloids, aldehydes, and phenols are examples of vegetal resources that can be effectively utilized to supply electrons for the generation of gold NPs by reduction of Au^+^ or Au^3+.^^137^ The reaction depends upon plant extract concentration, metal salt's presence, incubation duration, temperature, and reaction pH.

#### 4.2.2 Silver nanoparticles (NPs)

Silver NPs-based sensors have been targeted in different fields. The rapid advancement of technology in synthesizing silver NPs and their versatile, specific, and sensitive characteristics in various areas make it feasible to develop and utilize silver NPs-based sensors. Due to their consistent stability, favorable selectivity, and heightened sensitivity, silver NPs have been extensively regarded as an optimal candidate for superior on-site sensing capabilities. Silver NPs-based sensors have been more effectively utilized and, through their functionalization, have demonstrated better control over contaminants and rapid signal amplification than other nanostructured materials.^138^ Moreover, among all metals, silver NPs are regarded as the best thermal and electrical conductors. Silver NPs have been utilized in developing nanocomposite-based biosensors and are less expensive than gold NPs. Additionally, regarding ecologically friendly approaches, plant extracts, fungi, bacteria, and small biomolecules were employed as biological precursors in the biosynthesis of gold NPs, which emerged as a valuable alternative to chemical methods' drawbacks.^139^

#### 4.2.3 Platinum nanoparticles (NPs)

Platinum is a noble metal with a high melting point (1769 °C) and resists corrosion and chemical assaults. Platinum NPs in various morphologies, including nanocluster, nanotube, and cubic forms, serve multiple purposes as catalysts, electrodes, and nanosensors. Additionally, platinum NPs' high surface area-to-volume ratio makes them highly effective antibacterial agents.^140^ Platinum NPs are also generated through synthetic or biological means for application in the biomedical domain.^141^ The physical approaches encompass different processes that possess their advantages and disadvantages, such as laser ablation, evaporation and condensation, and solvothermal processes. The benefits include no toxic chemicals, high speed, uniform shape and size, and purity.

Conversely, the drawbacks encompass high energy consumption and cost, low productivity, reduced thermal stability, and substantial waste generation. Chemical methods involve plasma CVD, hydrothermal, polyol synthesis, pyrolysis, and the sol–gel process. These techniques necessitate the presence of metal precursors, stabilizing or capping agents, and reducing agents. The recognized precursors include PtCl_2_, Pt(NH_3_)_4_(NO_3_)_2_, and H_2_PtCl_6_, while the frequently employed reducing agents to control the size and shape of the NPs are ascorbate, trisodium, potassium bitartrate, and sodium borohydride.^140^ In addition, the biosynthetic pathway was employed using different plant extracts, such as those from *Pinus resinosa* and *Ocimum sanctum*, to synthesize platinum NPs biologically. The biosynthetic pathway has several benefits, including cost-reduction, tiny size (3–5 nm), non-toxicity, environmental friendliness, and quick synthesis. Still, it suffers from the difficulty of controlling the shape and size of the NPs. Various techniques can be utilized to characterize platinum NPs, such as TEM, XRD, SEM, and FTIR.

The numerous benefits associated with the distinct chemical and physical characteristics of metal NPs, further supported by their nanoscale size, explain their extensive use in nanosensors. Recently, noble metal NPs incorporated with carbon nanotubes (CNTs) have formed a novel type of composite materials. These materials effectively combine the distinct properties of noble metal NPs and CNTs, resulting in new functionalities arising from the cooperative effects between the two components. Consequently, noble metal NPs/CNTs nanohybrids have demonstrated remarkable potential in various fields, with particular emphasis on chemo/biosensors.^132^ For example, when silver NPs were used for immobilized graphene oxide (GO), colloidal carbon was coated on the NPs to improve their biocompatibility and eliminate toxicity.^142^

#### 4.2.4 Metal oxides

In addition to metal and CBNs, metal oxides (MOs) have garnered significant interest in creating chemical sensors.^143,144^ In addition to metal and CBNs, metal oxides (MOs) have garnered considerable interest in creating chemical sensors.^143,144^ Metal oxides include various materials that exhibit multiple characteristics, encompassing magnetic, dielectric, catalytic, and UV-absorption properties. To illustrate, iron oxide (Fe_3_O_4_) functions as a magnetic material with good biocompatibility that is valuable for separation and drug delivery. Since titanium is a plentiful and biocompatible material, titanium oxide (TiO_2_) NMs are frequently used as a supported material for decoration with other metal NPs in a variety of biomedical and biosensors applications as well as TiO_2_ serving as a potent photocatalyst.^145^ TiO_2_ NMs are also chemically stable with outstanding mechanical properties and huge surface area. Another category of metal oxide NPs is aluminum oxide NPs (Al_2_O_3_ NPs) that exhibit a wide range of biomedical and sensing uses due to their remarkable physicochemical characteristics, including their resistance to chemicals and wear as well as their extensive porous surface area, cost-effectiveness in preparation and ease of handling.^146,147^

**Fig. 8:**
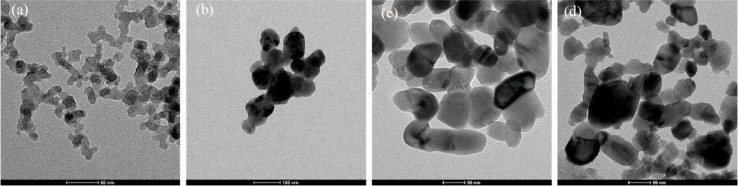
TEM images of metal oxides' nanoparticles: (a) (Al), (b) (Cu), (c) (Ti), (d) (Zn).^151^

Also, cerium oxide NPs (CeO_2_ NPs) play a significant role as oxidative catalysts, in addition to their use in chemical and humidity sensors.^148^ In contrast, chromium oxide NPs (Cr_2_O_3_ NPs) are used for electrochemical and strain sensing.^149,150^ These materials are cost-effective and stable, making them suitable for many intriguing applications. While most research on metal oxides has traditionally focused on industrial catalysis and ceramics, investigations into their interaction with biomolecules have also been explored. Numerous compelling sensors and biosensors involving different metal oxide NPs have emerged in recent years. The shape and size of various metal oxide NPs, as appeared under TEM, are shown in Fig. 8.^151^

### 4.3. Silicon-based nanomaterials (NMs)

Since silicon-based NMs have favorable biocompatibility, surface tailorability, excellent electrical, optical, and mechanical properties, and compatibility with conventional silicon technology, they are an essential class of NMs widely investigated.^152–154^ In particular, silicon nanowires (SiNWs) have demonstrated significant promise for various uses in sensors and bioimaging.^155,156^ SiNWs and SiNWs arrays have also been utilized recently to track environmental pollution. Three electrodes comprise the conventional field effect transistor (FET)-based silicon nanowire (SiNW) biosensor. Their charge density variation, which causes variations in the electric field at the SiNW's outside surface, is the mechanism underlying their sensing process.

Moreover, SiNW's exceptional qualities, including their tunable optical and electrical characteristics, high surface-to-volume ratio, and biocompatibility, made them attractive for viruses and metal ion detection sensors. Moreover, SiNWs-based electrochemical sensors can be enhanced to be available for quick and accurate pesticide detection. Bottom-up^157^ and top-down^158,159^ approaches are the most frequently used methods for the nanofabrication of SiNWs. SiNWs grow from a substrate in the bottom-up process. In contrast, a silicon-on-insulator fabrication technique is essential in the top-down method.

### 4.4. Semiconductor quantum dots

Recent developments in semiconductor quantum dots (QDs) and colloidal nanocrystalline semiconductors have demonstrated significant promise for technical and fundamental research applications.^160^ QDs are the perfect optical labels for sensing because of their unique photophysical characteristics, which include restricted emission bands, strong Stokes shifts, high fluorescence quantum yields, and very low photobleaching.^161^ Due to their size-controlled luminescent properties, QDs have demonstrated tremendous potential in molecular detection in recent breakthroughs. QDs have been used as optical markers for detecting heavy metal ions by researchers.^162–164^ One well-researched approach for sensing metal ions, pesticides, hazardous gases, tiny compounds, and industrial effluent uses fluorescent QD sensors.^165–168^ Chlorpyrifos (CP) and other organo-phosphorothioate pesticides are recognized explicitly by the fluorescence turn-on response, enabling pesticide detection at concentrations as low as 0.1 nM. Significantly, at a limit of 5.5 ppb, the fluorescence turn-on chemosensor can directly identify CP residues in apples.^169^

Due to their distinct size and shape-dependent optical properties, II–VI (CdSe, ZnS), III–V (InAs, InP), and IV–VI (PbS, PbTe) QDs have been researched over the past ten years. However, they are not environmentally friendly due to the inclusion of Pb and Cd elements, which limits their use.^170^ Thus, research into QDs with less hazardous and environmentally favorable components is necessary. In order to create less hazardous QDs with comparable exceptional optical qualities, I–III–VI ternary QDs are created by substituting one monovalent and one trivalent cation for the divalent cation found in II–VI binary QDs.^171^ The primary reasons for the increased interest in ternary I–III–VI QDs are their appealing optical and electrical characteristics, low toxicity, and environmental friendliness, providing a safer option than II–VI and IV–VI QDs.^172^ This makes them popular for biocompatible solar cell applications, water treatment, biological applications, and heavy metal ion detectors in water.^171^ These materials are considered one of the most recent varieties of QDs to be found and used in environmental and biological monitoring disciplines.

## 5. Sensing mechanisms of nanomaterials

NMs possess unique properties arising from their high surface-to-volume ratio, quantum confinement effects, and tailorable surface chemistry, making them attractive for sensing applications across various fields. The sensing mechanisms employed by NMs can be broadly categorized into optical, electrochemical, and other transduction principles, depending on the nanomaterial and the analyte of interest. Table 1 shows a range of nanomaterial sensors using different detection methods for highly sensitive analyte detection.

**Table 1:** Diverse nanomaterial-based sensing platforms exploiting various transduction mechanisms for high-sensitivity detection of analytes

Nanomaterial	Detection mechanism	Analyte	Detection range	Detection limit	Reference
Graphene quantum dots (GQDs) and acid-functionalized multiwall carbon nanotubes (MWCNTs)	Electrochemical	Dopamine	0.25–250 μM	95 nM	180
Gold and silver nanoparticles	Colorimetric sensing platform	Target DNAs and target proteins (thrombin and platelet-derived growth factor)	15–40 nM	19 nM	181
Graphene	Prism-coupled surface plasmon resonance (SPR) biosensor	Glucose	25–175 mg dl^−1^	—	182
Quantum dots	Electrochemiluminescence (ECL)	Alpha-fetoprotein (AFP) and carcinoembryonic antigen (CEA)	0.001–0.1 pg mL^−1^	0.4 fg mL^−1^	183
Graphene oxide	Fluorescent aptasensor	Aflatoxin B1	0 ng mL^−1^ - 3 ng mL^−1^	0.25 ng mL^−1^	184
Silica nanoparticles	pH-responsive fluorescent	pH	pH 5.5 to 9.0	—	185
Chitosan, graphene, and titanium dioxide (CS/RGO/TiO_2_)	Electrochemical sensing	Lead ions (Pb^2+)^	1 ng L^−1^ to 1000 ng L^−1^	0.33 ng L^−1^	186
Titanium dioxide nanotubes (TNTs) and silver nanoparticles (AgNPs)	Electrochemical biosensing	Heat shock protein 70 (HSP70)	0.1 to 100 ng mL^−1^	0.48 ng mL^−1^	187

### 5.1 Optical sensing mechanisms

- Localized Surface Plasmon Resonance (LSPR): noble metal nanoparticles, such as gold and silver, exhibit LSPR, which is the collective oscillation of conduction electrons induced by incident light. The LSPR frequency is highly sensitive to changes in the local dielectric environment, enabling the detection of analytes through shifts in the LSPR peak wavelength or intensity changes. Mayer and Hafner^173^ comprehensively reviewed recent advancements in localized LSPR, highlighting its significance in detecting molecular interactions near noble metal nanostructures. Their overview of LSPR sensing strategies, mainly focusing on metal nanoparticle-based sensors for label-free detection in biological applications, sheds light on the evolving landscape of LSPR technology and clarifies inconsistencies in nomenclature within the field.

- Fluorescence quenching or enhancement: semiconductor quantum dots exhibit size-dependent fluorescence properties, which the presence of analytes can modulate through quenching or enhancement mechanisms. Analyte binding or interaction with the quantum dot surface can alter the fluorescence intensity or lifetime, enabling sensitive detection. For instance, Das *et al.*^174^ studied the mechanism of fluorescence enhancement and quenching effect of single-walled carbon nanotubes (SWCNTs) on highly fluorescent graphene quantum dots (GQDs) over a wide range of SWCNT concentrations. At very low SWCNT concentrations, the fluorescence intensity of GQDs was enhanced. In contrast, systematic fluorescence quenching was observed at higher concentrations due to a combination of dynamic and static quenching, offering insights into fluorescence tuning for bio-imaging and drug delivery applications.

- Surface-enhanced Raman scattering (SERS): the SERS technique, renowned for its capability to detect molecular signatures through vibrational bonding information from Stokes shifted scattered photons, has found diverse applications in ultra-sensitive chemical sensing across various fields such as the food industry, explosive detection, forensic science, microbiology, medicine, and biomedical diagnostics, owing to its high detection sensitivity and the development of nanofabrication techniques leading to molecular detection limits down to single molecules, with plasmonic substrates being a major focus for such advancements. Plasmonic nanostructures, such as metal nanoparticles or nanostructured surfaces, can enhance the Raman scattering signal of analytes adsorbed on their surfaces by several orders of magnitude. This enhancement enables the detection of low concentrations of analytes through their unique Raman fingerprints. Recently, Mandal and Tewari^175^ provided a comprehensive review that primarily focuses on the progress made in SERS over the last 20 years, emphasizing SERS substrate fabrication techniques and chemical sensing, with additional discussions on its applications in food safety, food, and fuel adulteration, forensic science, defense, biology, and biomedical diagnostics.

### 5.2 Electrochemical sensing mechanisms

- Electrical conductivity changes: carbon nanotubes, graphene, and other conductive NMs can exhibit changes in their electrical conductivity upon adsorption or interaction with analytes. These changes can be measured and correlated with the analyte concentration, enabling the development of chemical sensors and biosensors. For instance, Li *et al.*^176^ investigated the development of a simple, reliable, and reproducible single-walled carbon nanotube (SWNT) sensor platform for gas and organic vapor detection at room temperature. The study demonstrated well-defined and reproducible linear electrical responses with a detection limit of less than 44 ppb for NO_2_ and 262 ppb for nitrotoluene. Moreover, Shooshtari *et al.*^177^ investigated the effect of humidity on the electrical conductivity of vertically aligned carbon nanotube (CNT)-based gas sensors designed for volatile organic compound detection. The study found that an increase in relative humidity from 10% to 80% resulted in a 4% reduction in sensor conductivity, while conductivity slightly increased above 80% humidity.

- Catalytic properties: metal nanoparticles, such as platinum, palladium, and gold, possess catalytic properties that can facilitate electrochemical reactions involving analytes. The presence and concentration of analytes can be determined by monitoring the electrochemical response, such as current or potential changes. For instance, Zhou *et al.*^178^ developed a novel electrochemical sensor for formaldehyde detection by depositing nanostructured platinum-palladium alloy onto a Nafion-coated glassy carbon electrode. This sensor demonstrated remarkable electrocatalytic activity, with a linear detection range of 10 μM to 1 mM and a low detection limit of 3 μM in acidic solution, showing promise for various applications.

- Ion-gating effects: transistor-based sensors employing semiconducting NMs, such as carbon nanotubes or metal oxide nanostructures, can leverage ion-gating effects. The binding of charged analytes to the nanomaterial surface can modulate the electrical properties, enabling the detection of various ionic or charged species. Subramanian *et al.*^179^ investigated the potential of titanium dioxide (TiO_2_) for next-generation transistors, particularly ion-gated transistors (IGTs). In their work, TiO_2_ films were fabricated through a wet chemical approach, demonstrating transistor behavior with room temperature ionic liquids, aqueous electrolytes, and pH sensing capability in TiO_2_ IGTs, boasting a ∼48 mV pH^−1^ sensitivity. Furthermore, they showcased the viability of low-temperature, solution-processed TiO_2_-based IGTs on flexible PET substrates, which exhibited stability even under moderate tensile bending.

### 5.3 Other sensing mechanisms

- Mechanical transduction: NMs can be incorporated into mechanical transducers, such as cantilevers or resonators, where the mass or surface stress changes induced by analyte binding can be detected through shifts in resonance frequency or cantilever deflection.

- Magnetic property changes: magnetic nanoparticles, such as iron oxide or ferrite nanoparticles, can change their magnetic properties upon interaction with analytes. These changes can be measured using magnetic sensors or techniques like magnetic resonance imaging (MRI).

- Thermal or calorimetric effects: some NMs can exhibit thermal or calorimetric responses upon binding or reacting with analytes. By monitoring temperature changes or heat flow, the presence and concentration of analytes can be determined.

It is worth noting that the choice of sensing mechanism and the specific nanomaterial employed depends on the target analyte, the desired sensitivity and selectivity, and the application requirements. Researchers often combine various NMs and sensing mechanisms to develop robust and versatile sensing platforms tailored to specific analytical needs.

## 6. Application of nanomaterial-based nanosensors

### 6.1. Overview

Nanosensors have numerous advantages over traditional sensors made from bulk materials. Their sensitivity and specificity are enhanced due to unique physicochemical characteristics at the nanoscale. NMs' high surface-to-volume ratio and novel properties, such as nanophotonics, enable increased sensitivity and specific detection. Moreover, nanosensors offer cost and response time advantages, making them suitable for high-throughput applications. They provide real-time monitoring compared to conventional methods like chromatography or spectroscopy, which can be time-consuming and require extensive sample preparation. With applications in healthcare monitoring, environmental monitoring, and various other fields, nanosensors are paving the way for advancements in personalized medicine, disease detection, and scientific research, as shown in Table 2.^1,68,188–191^

**Table 2:** Some properties of NMs used in nanosensors

Nanosensors	Property	References
Carbon nanotubes (CNTs)	Highlighted for their high electron transferability, extensive level of detection, ability to stabilize enzymes, and sensitivity to degradation by white blood cells	192–194
Graphene quantum dots	Noted for improving the surface and electrochemical properties of sensors when combined with other nanostructures	195 and 196
Magnetic NPs	Discussed their ability to enhance catalytic properties and glucose detection due to the activity of enzyme glucose oxidase when bound to copper. They are also mentioned for their magnetic reusability and selectivity	197–199
Nano optical sensor	Mentioned for their role in noninvasive glucose monitoring and the modulation of release rates in response to abdominal deformities	200 and 201
Electrochemical biosensors	Characterized by the ability to quantify two different mechanisms of ion exchange within the sensor and their contribution to the diversity of biosensor fabrication	202 and 203
Visual nanosensor	Described as the most advanced type, with a small volume yet powerful detection capabilities and their ability to function optimally even with low concentrations of analytes	204–206

### 6.2. Application of nanomaterial-based nanosensors in healthcare

Nanomaterial-infused nanosensors have emerged as a pivotal innovation in healthcare and biomedical fields, demonstrating remarkable proficiency in identifying tumor-specific biomarkers, circulating tumor cells, and extracellular vesicles discharged by tumors. This capability facilitates intelligent cancer detection, significantly enhancing patient prognosis.^207^ An array of NMs, including gold nanoparticles, quantum dots, polymer nanoparticles, carbon-based NMs, and metal/metal oxide nanoparticles, has been rigorously explored for their potential in biosensing endeavors.^208^ Moreover, the advent of nanomaterial-based flexible sensors (NMFSs) represents a significant leap forward. These sensors, capable of adhesion to human skin or incorporation into garments, are designed to track physiological metrics and furnish vital medical insights.^209^ Specifically, carbon-based NMs such as carbon nanotubes, graphene, carbon quantum dots, and carbon fibers have been employed as sensor elements for biomarker detection.

In contrast, other materials, such as biodegradable silk nanofibers, have been employed to harvest energy to power these sensors.^210^ These materials are distinguished by their high surface-to-volume ratio, exceptional electrical conductivity, and compatibility with biological systems.^211^ Collectively, these advancements underscore the transformative impact of NMs in the biosensor domain, enabling swift and accurate detection, continuous health monitoring, enhanced sensitivity, and cost-efficiency in healthcare and biomedical applications.^212^

**Fig. 9:**
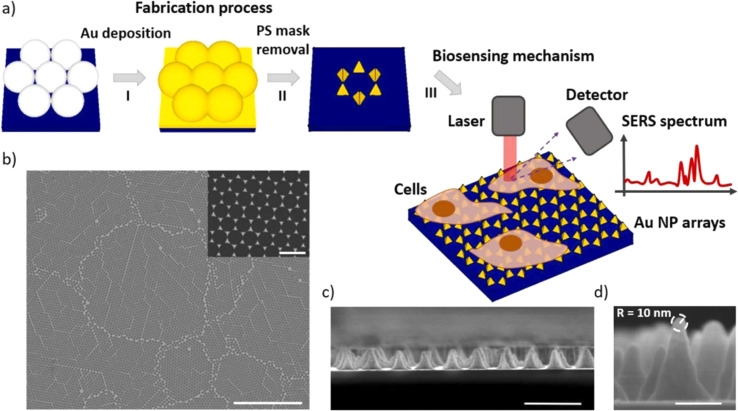
Illustrates (a) the construction sequence of the gold nanoparticle (Au NP) array on glass, serving as an SERS platform to investigate cellular activities. This process involves assembling a colloidal mask, depositing gold, and then removing the nanospheres. Subsequently, the Au NP arrays function as a SERS foundation for tracking the amplified Raman signals of neurons throughout various phases of their differentiation. (b) A scanning electron microscope (SEM) image displays the extensive layout of the Au NP array (scale bar: 10 μm), with an inset providing a closer look at the Au NPs to showcase their periodic arrangement (scale bar: 500 nm). (c) A cross-sectional SEM view reveals the vertical structure of these nanoforms (scale bar: 500 nm). (d) An enhanced view of the cross-section SEM image of an individual Au NP, emphasizing the acuteness of its tip, which measures 10 nm in curvature (scale bar: 100 nm).^213,214^

In the study presented in Fig. 9 by M. Iarossi *et al.*,^213^ the team innovatively crafted a surface-enhanced Raman spectroscopy (SERS) active interface using an array of gold nanopyramids (Au NPs) equipped with plasmonic tips. This cutting-edge technique was designed to observe cellular activities and effectively distinguish between undifferentiated and differentiated neurons through the application of principal component analysis. The gold nanopyramids, characterized by tip curvatures measuring 10 nm and exhibiting localized plasmon resonance at a wavelength of 785 nm, were utilized to examine ND7/23 neurons non-invasively. The collection of SERS spectra from cells situated on these gold nanopyramids, conducted with a Raman spectrometer, unveiled the presence of critical cellular components such as membrane constituents, proteins, lipids, and nucleic acids like DNA and RNA. Following the SERS analysis, a cell viability test confirmed that the majority of the cells remained viable, with only a minimal number of them perishing on the gold nanoparticle arrays placed atop glass surfaces.

M. K. Hameed *et al.*^215^ developed anisotropic gold nanostars (AuNS) by utilizing varying amounts of silver nitrate (AgNO_3_) to guide their shape, thereby adjusting the length of the spikes on the nanostars. The uptake of these AuNS by MDA-MB-231 breast cancer cells was notably more effective than in normal cells, showcasing their promise as carriers for cancer treatment drugs. The shape of the AuNS was determined by the levels of gold(iii) chloride and the AgNO_3_ additive, with the nanostars formed without the use of surfactants displaying distinct characteristics from those synthesized with surfactants. Confocal microscopy images provided evidence of the AuNS being absorbed by the cancer cells, marked by strong blue fluorescence within the nuclei and green fluorescence in the cytoplasm. This research underscores the critical role of nano spike morphology in enhancing the SERS analysis of cell components, underlining its potential benefits in biomedical applications.

**Fig. 10:**
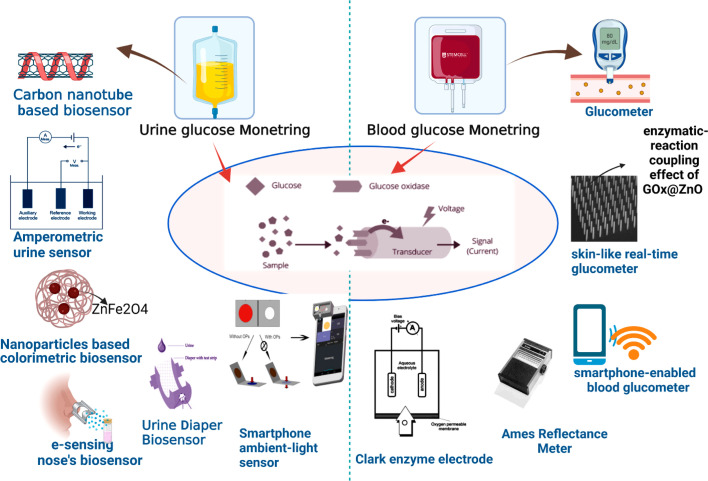
Diagnosis of glucose level in urine and blood with the help of modern biosensor tools.^214,216^

The vigilant surveillance of glucose concentrations within the urine is paramount, as heightened levels are harbingers of diabetes mellitus progression. A urinalysis yielding a positive outcome signifies that the glucose concentrations within the organism surpass the threshold of 50–100 mg dL^−1^ (equivalent to 2.78–5.55 mM). The pioneering glucose meter for urine in Japan unveiled in 1996, demonstrated commendable proficiency in monitoring glucose concentrations spanning from 0 to 500 mg dL^−1^. Subsequently, in 1999, the TOTO Corporation spearheaded the innovation of a glucose monitoring device seamlessly integrated into toilet seats, thereby facilitating the analysis of diluted urine specimens, as depicted in Fig. 10. Further, the exploration into carbon nanotubes (CNTs) for the detection of glucose in urine has been noteworthy. The strategic employment of CNTs dissolved in an aqueous solution of the biopolymer chitosan (CS) has enabled precise glucose measurements in urine, devoid of any interference, with a detection threshold reaching as low as 3 M.^216^

T. Patil *et al.*^217^ highlighted the distinctive physicochemical characteristics of gold nanoparticles (AuNPs), noting their potential in various biological contexts. Various synthesis techniques, including biologically based ones, can produce AuNPs. The surface functionalization process grants these nanoparticles colloidal stability, broadening their utility in water-based and physiological settings. Particularly, the surface plasmon resonance (SPR) feature of AuNPs is leveraged in developing biosensing and bioimaging tools for diagnostic purposes and pathogen identification. Applications of functionalized AuNPs span bioimaging, biosensing, cancer treatment, drug delivery, hyperthermia treatment, and fighting microbial infections. There is, however, a call for more research into the attachment of biomolecules to the nanoparticle surface to refine the use of AuNP-based diagnostic technologies. Additionally, the issue of how these nanoparticles are cleared from the kidneys in live-body medical applications presents a significant challenge that needs further investigation.

### 6.3. Application of nanomaterial-based nanosensors in environmental monitoring

#### 6.3.1 Unparalleled detection of pollutants

Nanosensors, specifically nanomaterial-based nanosensors, offer numerous advantages over traditional sensors in detecting pollutants for environmental monitoring. They are cost-effective, susceptible, and possess excellent detection potential and selectivity. Nanosensors have successfully detected contaminants such as ethanol and specific contaminants like 2,6-dimethoxy phenol.^1,191,218^ In agriculture, nano biosensors can monitor parameters like temperature,^219^ humidity,^220^ and soil components to reduce water usage and minimize chemical use at specific times and locations. They have also shown promise in detecting organic pollutants in agriculture with high accuracy and sensitivity. Nanosensors can also transform environmental monitoring by providing real-time air and water quality information. They can detect changes in chemicals found in water, soil, and the environment, aiding in identifying pollutants and toxins. Developing nanosensors using nanotechnology offers unparalleled precision, sensitivity, and selectivity, revolutionizing sensor fabrication. While there are concerns about health and environmental impacts, a collaboration between scientists and policymakers is necessary to ensure that the benefits outweigh potential risks. Nanosensors can potentially improve various industries worldwide by enhancing detection capabilities and improving overall efficiency and accuracy.^1,191,218^

#### 6.3.2 Guiding remedial strategies

Nanosensors have emerged as competent tools in environmental monitoring, distinguishing themselves through their ability to detect a wide range of pollutants, including those in minute concentrations. By harnessing nanobiocatalysis, these sensors achieve enhanced specificity for particular contaminants, thereby elevating the precision of detection efforts. Their application extends across a spectrum of environmental pollutants, encompassing pesticides, heavy metals, bacteria, antibiotics, and various organic substances. Innovations have led to the creation of graphene-based nanohybrid aptasensors designed to detect these pollutants in diverse mediums such as water, soil, and other environmental samples. The ongoing research and development in this arena promise to further refine sensor capabilities, paving the way for novel detection methodologies and bolstering efforts to safeguard the environment.^1,189,218,221,222^

Y. Shimizu *et al.*^223^ conducted a study on the SO_2_ detection capabilities of various semiconductor metal oxides, identifying WO_3_ as having the highest sensitivity at 400 °C. They discovered that incorporating 1.0 wt% Ag notably enhanced this sensitivity at 450 °C. This improvement is attributed to changes in the surface states of SO_2_-related adsorbates and the electronic interactions between the adsorbates and WO_3_. The study highlights how adding noble metals to WO_3_ can significantly improve its ability to sense SO_2_, making it highly applicable in environmental monitoring systems to detect sulfur dioxide concentrations in the atmosphere. Such sensors are particularly valuable in industrial settings, power generation plants, and other operations where it is essential to monitor and control SO_2_ emissions to safeguard environmental quality and public health. Moreover, these enhanced sensors can be incorporated into air quality monitoring networks, offering timely and accurate data on pollution levels.

Q. Wang *et al.*^224^ synthesized the gas-sensing properties of In–Sn oxides composites by hydrothermal method. This study explores the successful synthesis of nanoparticles using an environmentally friendly approach and investigates their gas-sensing performances towards ethanol. The findings contribute to developing advanced materials for gas sensors with improved sensitivity and selectivity, which are crucial for various industrial and environmental monitoring applications.

### 6.4 Application of nanomaterial-based nanosensors in industries

#### 6.4.1 Ensuring process safety

Nanosensors have the potential to revolutionize various sectors, including industries, by ensuring process safety. These nanoscale devices can measure physical quantities and convert them into detectable and analyzable signals. With the unique properties associated with NMs, such as their large surface area and surface reactivity, nanotechnology allows for realizing previously impossible technologies with bulk materials.^1^

One of the main advantages of nanosensors is that they offer precise detection capabilities for sensing physical parameters, CBRN (chemical, biological, radiological, and nuclear) agents, and more. Additionally, nanosensors are tiny and portable, making them suitable for high-throughput applications. In industries, nanosensors can be integrated with nanoelectronics to add native processing capability, enabling real-time monitoring and control. For example, nanosensors can detect product defects early in manufacturing processes, allowing for adjustments before large quantities are produced.^1,68,189,221,225^ This not only saves time but also reduces costs.

Furthermore, nanosensors have significant applications in the aerospace and defense industries. In aerospace applications, they enable real-time structural monitoring to ensure the integrity of aircraft components and minimize maintenance costs. Nanosensors also improve fuel efficiency and flight safety while reducing environmental impact.

In the defense sector, nanosensors offer advanced threat detection and surveillance capabilities. They can detect CBRN threats, providing crucial situational awareness and protecting military personnel. Additionally, nanosensors find applications in explosive detection, camouflage technology, and unmanned aerial vehicles (UAVs) for surveillance.

Moreover, nanosensors can be used for environmental monitoring in industries such as water quality assessment. By effectively identifying chemicals and bacteria that threaten aquatic life and human health, these sensors ensure the safety of water resources.^1,68,189,221,225^

#### 6.4.2 Optimization of processes

The optimization of processes through the application of nanomaterial-based nanosensors has the potential to revolutionize multiple sectors, including industries. Nanosensors are nanoscale devices that can measure physical quantities and convert them into detectable and analyzable signals. They offer high sensitivity, good selectivity, small size, and portability, making them suitable for various applications.

In industries, nanosensors have proven to be invaluable tools. They can be used to monitor the quality of food and pharmaceuticals, ensuring that they meet the required standards. Additionally, nanosensors are crucial in safeguarding workers in dangerous work settings by detecting potential hazards.

One of the key advantages of nanosensors in industries is their ability to enhance efficiency while reducing costs and minimizing waste. For example, in manufacturing, nanosensors can detect product defects early, allowing adjustments to be made before large quantities are produced.^1,68,189,221,225^ This not only saves time but also saves companies money.

A remarkable development in nanosensors is the creation of self-powered nanosensors called triboelectric nanosensors (TENS). These sensors use an array of mercury-sensitive tellurium nanowires to detect trace amounts of mercury ions in water or food and provide instant reports on their presence. This breakthrough demonstrates the potential of nanosensors in monitoring processes and ensuring product safety.

In the electronics industry, the integration of nanosensors is transforming the field. These sensors offer enhanced precision, sensitivity, and functionality for consumer electronics, communication devices, and smart systems. Nanosensors enable faster data processing, improved energy efficiency, and miniaturization of electronic components, paving the way for advanced devices and technologies.

Moreover, in the manufacturing sector specifically, nanosensors play a vital role in quality control and process optimization. They enable real-time monitoring of production parameters to ensure optimal performance and cost-effectiveness. Nanosensors also contribute to developing smart factories and implementing Industry 4.0 principles, increasing productivity and efficiency.^1,68,189,221,225^

### 6.5 Application of nanomaterial-based nanosensors in security and defense

In the fields of forensics, environmental monitoring, and the production and storage of explosives, the ability to detect minute quantities of explosives is crucial. The threat that explosives pose to both national and global security remains significant. Recent incidents have highlighted the essential role of explosive trace detection (ETD) technologies.^226^ The use of homemade explosives (HMEs) in terrorist activities is especially concerning due to their high potential for destruction, the ease of acquiring materials, and the availability of online instructions for their creation and use. Therefore, improving current ETD techniques and continuously exploring new methods is critical.

**Fig. 11:**
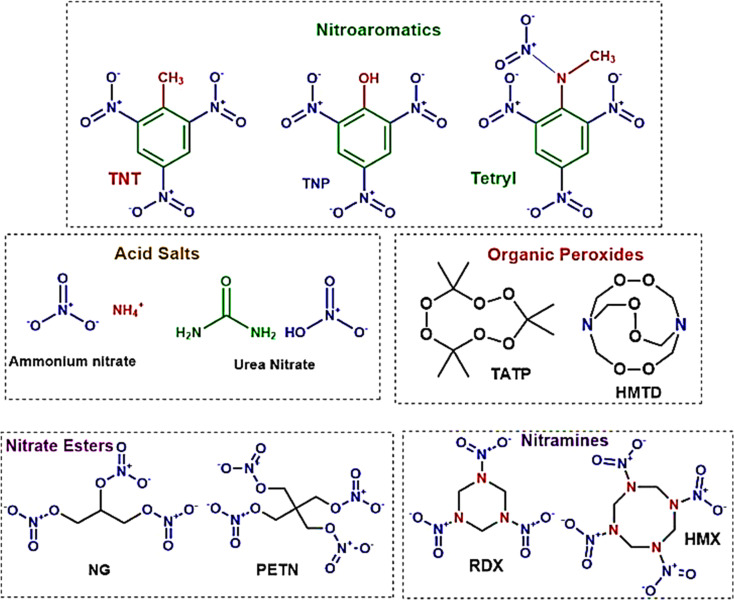
Provides a systematic categorization of explosives based on their chemical groupings. This diagram serves as a comprehensive framework for understanding the diverse classifications within the field of explosives, structured according to their distinct chemical compositions.^228^

Explosives are classified according to their molecular structures and compositional characteristics, as depicted in Fig. 11. The use of nitroaromatic compounds, such as TNT (trinitrotoluene) and DNT (dinitrotoluene), in ammunition raises environmental concerns.^227^ Organic peroxides, which are notably sensitive to heat, shock, and friction, are commonly used in the making of homemade explosives (HMEs). Nitrate esters, typically liquid, are employed in the military for purposes such as plastic explosives, detonators, and propellants. Ammonium nitrate/fuel oil (ANFO) mixtures and urea nitrate (UN) are among the most frequently used materials for crafting improvised explosive devices (IEDs).

The wide range of chemical and physical attributes of explosives complicates collecting samples and detecting traces of these materials. Typically, particulate sampling is conducted with sampling wands, where the direction, pressure, and method applied are critical to collect samples efficiently. Vapor sampling poses an additional challenge due to many explosives' shallow vapor pressures. Furthermore, the “sticky” nature of numerous explosives causes them to cling to various surfaces, leading to the accumulation of explosive particles and thus reducing the amount of analyte molecules available per sample volume. Examining luggage and cargo, often wrapped in protective materials, presents logistical and operational challenges that can hinder effective sampling. In every instance, there is a risk that non-explosive materials could cause false positive alerts. Therefore, adopting novel detection technologies requires a comprehensive assessment of regulatory (concerning threats, sensitivity, selectivity), operational, civilian, and economic factors.

**Fig. 12:**
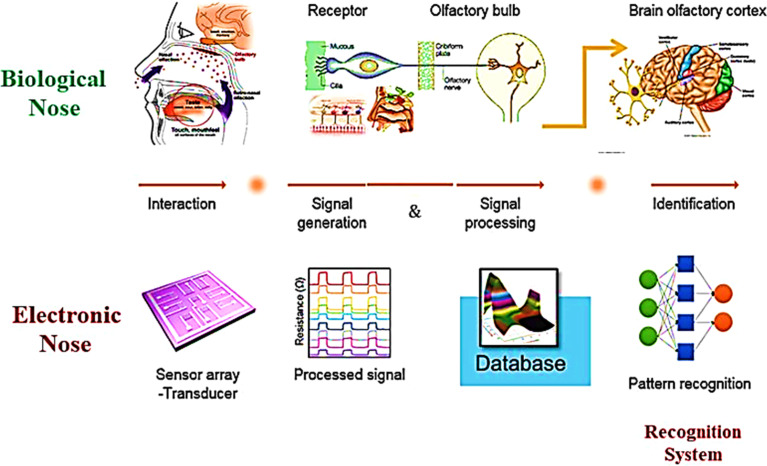
Delineates the concept of the Electronic Nose (E-nose), an innovative diagnostic tool designed to mimic the olfactory system's functionality. This illustration encapsulates the E-nose technology's principle operations and theoretical underpinnings, highlighting its pivotal role in detecting and analyzing complex aromas and chemical signatures.^228^

Investigations and advancements within the NMs sphere have illuminated nanostructured entities' potential to serve as detectors for various chemical and biological substances, including explosives. Developing exceptionally diminutive apparatuses endowed with heightened detection capabilities is a cornerstone of nanosensor technology. Notably, the concepts of electronic noses, nanowire/nanotubes, and nanomechanical systems emerge as the most promising candidates poised to establish effective technological frameworks for identifying trace amounts of explosives. The electronic nose approach endeavors to replicate the olfactory capabilities of bomb-detection canines, albeit devoid of their associated limitations. This innovative device integrates a chemical sensing mechanism, a sample collection module, and a pattern recognition apparatus, typically in the form of an artificial neural network, as shown in Fig. 12, to achieve its function.^228^

**Fig. 13:**
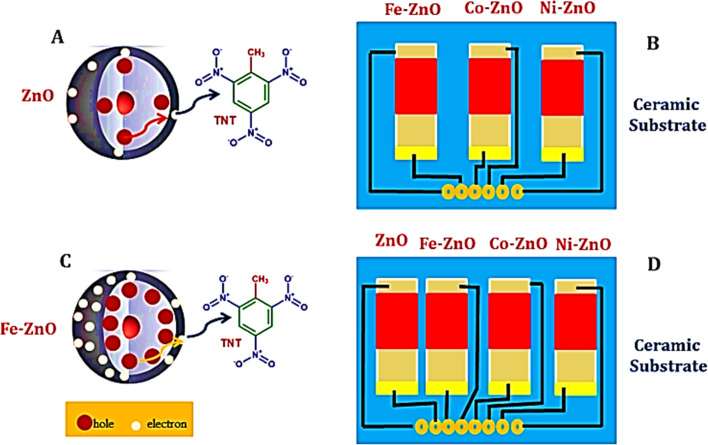
Depicts the Fe-doped ZnO chemiresistive sensor configuration, providing an insightful visualization of the intricacies of charge transfer mechanisms within both undoped and doped ZnO structures (A and C). Furthermore, it offers a schematic overview of a gas sensor array, which integrates three doped sensors—namely Fe–ZnO, Co–ZnO, and Ni–ZnO (B), alongside a comprehensive representation of an expanded array incorporating an additional undoped ZnO sensor, thereby culminating in a four-sensor array that includes ZnO, Fe–ZnO, Co–ZnO, and Ni–ZnO (D). This figure effectively conveys the enhanced detection capabilities facilitated by the strategic incorporation of dopants into ZnO matrices.^228^

L. D. Bastatas *et al.*^229^ designed a chemiresistive sensor to detect ammonium nitrate using silica nano springs coated with zinc oxide (ZnO). This breakthrough leverages the enhanced activation of charge carriers for more efficient desorption of analytes and boosts sensor recyclability through UV light irradiation. J. Qu *et al.*^230^ also developed a chemiresistive sensor array to identify various explosives, including TNT, DNT, and TNP. By doping ZnO with metals like cobalt (Co), nickel (Ni), and iron (Fe), they were able to enhance the adsorption of explosive molecules on the metal oxide (MO_*x*_) surface, significantly increasing the sensor's sensitivity. These enhancements allowed for detecting explosives at concentrations ranging from parts per billion to parts per trillion, with a rapid response time of just 12 seconds. The enhanced speed of these doped sensors is credited to the reduced distance for charge transfer between the charge reservoir layer and the surface defect centers on the MO_*x*_ nanoparticles, as demonstrated in Fig. 13.

The development of some MO_*x*_ (metal oxide) sensors that can function at lower temperatures, as showcased by L. Bastatas and J. Qu, marks a significant improvement, offering potential benefits such as reduced energy consumption and decreased risk of hazards in their application. By adjusting the operational temperature, employing arrays of sensors, and using composite materials, the sensitivity of MO_*x*_ semiconductors towards specific explosive compounds can be significantly enhanced. The rate at which oxygen is adsorbed onto the surface of MO_*x*_ changes with temperature variations, allowing for identifying particular gases through the sensor's response. Leveraging data from multiple sensors, each operating at different temperatures can increase selectivity in detecting compounds. Furthermore, composite MO_*x*_ structures can be custom-designed, selecting component materials specifically for their efficacy in sensing distinct gas analytes, thus refining the detection process.^231,232^

J. Warmer *et al.*^233^ employed sensors based on tin dioxide (SnO_2_) and tungsten trioxide (WO_3_) to detect triacetone triperoxide (TATP) and diacetone diperoxide (DADP). By capitalizing on the temperature-dependent oxidative and reductive properties of organic peroxides, they improved the selectivity of WO_3_ films for TATP through temperature adjustments. The detection threshold for these sensors was determined to be in the parts per billion (ppb) range, showcasing their high sensitivity in identifying these specific explosives.

Z. Yang *et al.*^234^ developed an innovative array of Schottky optoelectronic sensors by combining silicon nanowires (SiNWs) with titanium dioxide (TiO_2_) and reduced graphene oxide, achieving impressive detection limits for various nitro-based explosives from as low as 0.05 parts per quadrillion (ppq) to 74 parts per billion (ppb). In further research, by replacing TiO_2_ with zinc oxide (ZnO), this group enhanced their sensor array's ability to differentiate between urea, black powder, and a range of nitrate- and nitro-based explosives.^235^ This progress highlights the importance of utilizing the catalytic properties of certain metal oxides to create thermodynamic sensors that offer selective detection capabilities. This method typically involves measuring the electrical energy needed to keep a constant temperature across two microheaters, one of which is coated with a catalytic material. This setup aids in gathering thermodynamic data that reflects the unique catalytic activity of the analyte.^236^

M. Amani *et al.*^237^ utilized tin dioxide (SnO_2−*x*_) and ZnO to detect triacetone triperoxide (TATP) at parts per million (ppm) levels, drawing on the catalytic qualities of these oxides to inform their sensor design. A. S. Rossi *et al.*^238^ further improved this technique, achieving detection at the parts per billion (ppb) level by employing sensors with reduced thermal mass. Their research established a sensitivity ranking among metal oxide catalysts towards TATP, finding that SnO exhibited higher sensitivity than ZnO, which was more sensitive than copper oxide (CuO). This hierarchy of sensitivity is linked to the catalytic oxidation or reduction processes triggered by the decomposition of TATP.^239^ Enhancements in this field also included incorporating a conductometric platform, enabling the simultaneous collection of both thermodynamic responses and electrical resistance measurements from the catalytic metal oxide (MO_*x*_) sensor, thus providing a more comprehensive analysis.

The study of graphene and carbon nanotubes (CNTs) for use in Explosive Trace Detection (ETD) has drawn significant interest due to their superior electrical, mechanical, and chemical qualities over traditional inorganic materials.^240^ These carbon-based semiconductors, which can be chemically functionalized, are compatible with economical printing processes.^241,242^ Graphene, with its two-dimensional configuration offering a large surface area to volume ratio and a sp^2^ network, excels in detecting electron-deficient nitroaromatic compounds. Enhancing sensitivity to these compounds involves adding oxygen-containing functional groups, as found in graphene oxide^243^ or introduced during production,^244^ which strengthens van der Waals forces with NO_2_ groups.

Techniques to improve graphene's selectivity for ETD have included peptide modifications, nanoparticle incorporation, doping,^245^ adding organic polymers, and further peptide adjustments.^246,247^ Research has primarily focused on nitroaromatic compounds, yet the remarkable conductive properties of both single-walled (SW) and multi-walled (MW) CNTs have been explored for explosives sensing. Studies by Woods and Star have highlighted the critical role of charge-transfer and π-stacking interactions between CNTs and nitroaromatic explosives,^248,249^ indicating the vast potential of these carbon-based materials in advancing ETD sensor technology.

**Fig. 14:**
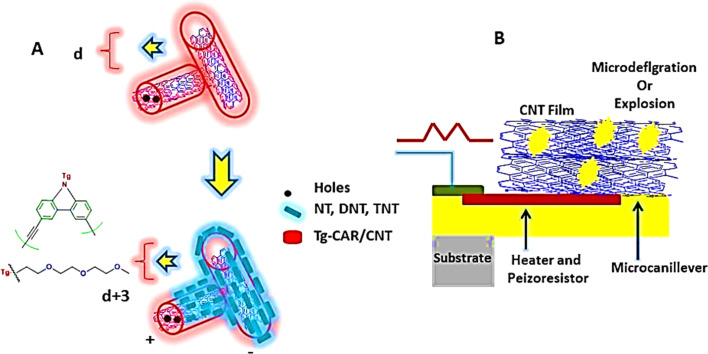
Illustrates the detection mechanisms for nitroaromatic explosives utilizing carbon nanotubes: (A) features single-walled carbon nanotubes (CNTs) that have been functionalized with Tg-Car/CNTs, and (B) showcases a piezoelectric microcantilever sensor enveloped in a CNT film. This figure delineates the application of advanced carbon nanotube technology in the sensitive and selective sensing of explosive materials.^228^

In the field of chemiresistive sensors for detecting trace vapor levels of nitroaromatic explosives, an innovative method has been introduced, utilizing a porous, thin film of single-walled carbon nanotubes (CNTs) coated with a carbazolylethynylene oligomer. These sensors excel in identifying trace amounts of 2,4,6-trinitrotoluene (TNT), 4-nitrotoluene (NT), and 2,4-dinitrotoluene (DNT) vapors, achieving sensitivity in the parts per billion (ppb) to parts per trillion (ppt) range.^250^ Remarkably, these sensors maintain outstanding selectivity for NT even at much higher vapor concentrations compared to other common organic substances, as illustrated in Fig. 14. In related research, J. S. Stefano *et al.*^251^ explored the performance of a TNT electrochemical sensor featuring electrodes coated with either pure or acid-treated CNTs. Their results reveal that electrodes with residual metallic impurities in pure CNTs enhance the electrochemical detection of TNT, a phenomenon credited to the catalytic actions and increased surface roughness of the electrodes.

The arsenal for explosive trace detection (ETD) is broadened with the inclusion of carbon dots, a form of nano-carbon distinguished by its fluorescent properties and chemical stability.^252^ M. Wang *et al.*^253^ have crafted a magnetic carbon dot-based molecularly imprinted polymer (MIP) composite engineered for detecting trinitrophenol (TNP) with a detection limit of 0.5 nM. In another development, B. Ju *et al.*^254^ utilized a straightforward solvothermal method to create carbon dots from *o*-phenylenediamine and chloroform. A colorimetric sensor employing these carbon dots demonstrated a detection limit of 2 μM. These carbon dots were fixed onto ordinary filter paper, illustrating a real-world application proving the viability of using this sensor technology in various environments.

Filanovsky *et al.*^255^ investigated the use of modified carbon electrodes, augmented with mesoporous titanium dioxide as a base, with ruthenium, platinum, or gold nanoparticles deposited on top for TNT detection. The use of TiO_2_–PtNP and TiO_2_–AuNP electrodes brought significant benefits, such as the clear separation of TNT from oxygen reduction signals, increased sensitivity, and a stable linear correlation between peak currents and TNT levels. In another study, M. Riskin *et al.*^256^ utilized gold nanoparticles (AuNPs) to enhance the sensitivity of electrochemical detection techniques for TNT. They developed a functionalized electrode that could detect TNT at parts per trillion levels by attaching AuNPs to an Au electrode and interconnecting them with oligoaniline chains. This configuration, optimized by adjusting the donor–acceptor interactions between TNT and the donor-linked AuNPs, also included molecular recognition sites within the oligoaniline-linked AuNPs, further boosting sensitivity to detect TNT concentrations as low as 200 picomolar (pM).

**Fig. 15:**
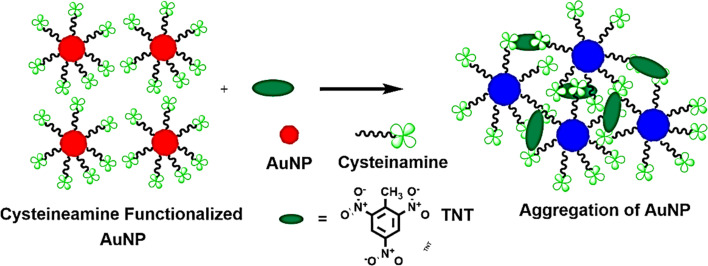
Presents a schematic depiction of the direct colorimetric method for detecting TNT, elucidating the electronic interactions between cysteamine and TNT. This illustration captures the fundamental principles underpinning the colorimetric response triggered by these interactions.^228^

Y. Jiang *et al.*^257^ developed a colorimetric technique for detecting TNT at the picomolar level, utilizing gold nanoparticles in a streamlined approach. This method hinges on the color shift of AuNPs, prompted by the donor–acceptor interactions between TNT and cysteamine. Cysteamine serves a dual role: it acts as a primary amine and stabilizes the AuNPs. When TNT is introduced into the solution, the cysteamine-stabilized AuNPs cluster together, leading to a color transition from red to violet blue, as illustrated in Fig. 15. The Jiang^257^ group noted a discernible color change visible to the naked eye when adding 114 pg L^−1^ of TNT to the mixture.

Surface-enhanced Raman spectroscopy (SERS) is recognized as an exceptionally sensitive method for detecting molecular entities, including trace amounts of explosives.^258^ S. S. R. Dasary *et al.*^259^ showed that when modified with cysteine, gold nanoparticles serve as efficient SERS probes for detecting TNT in water at concentrations as low as 2 picomolar (pM). This detection capability is attributed to forming the Meisenheimer complex between TNT and cysteine on the gold nanoparticles, creating “hot spots” that significantly boost the Raman signal. Enhancing this technique, L. Yang *et al.*^260^ pushed the boundaries of SERS-based TNT detection by employing silver nanoparticles functionalized with *p*-aminothiophenol (PATP) and encapsulated on silver molybdate nanowires. The PATP molecules on the silver nanoparticles undergo a catalytic coupling reaction to form dimercaptoazobenzene (DMAB), establishing imprint molecule sites that act as SERS “hot spots” and significantly intensify the Raman signal. This approach's detection limit for TNT was pinpointed at around 10 femtomolar (fM).

## 7. Applications of machine learning in the nanosensors field

As mentioned earlier, nanomaterial-based sensor platforms are becoming up-and-coming tools for versatile and ultra-sensitive detection.^261–263^ Their appeal lies in finely tuning optical, electrical, and electrochemical properties, enabling fast readouts, portability, and ease of use.^264,265^ However, several challenges must be addressed to translate nanosensor technologies into practical applications.^1,266^ These challenges include detecting analyte concentrations at ultralow levels (down to parts per billion or nanomolar levels), coping with complex sample matrices containing numerous interfering species, addressing issues related to differentiating isomers and structural analogs, and managing intricate, multidimensional datasets.^267,268^ Advanced artificial intelligence techniques, including machine learning (ML), could help to boost the performance of this kind of sensors for medical applications,^269^ nanotoxicology,^270^ neural prosthesis,^271^ wireless technology,^272^ smart agriculture,^273^ environmental monitoring,^274^ and advanced medical manufacturing technologies.^275^

ML is a pivotal category within the realm of informatics, offering methodologies that enable machines to learn from data autonomously. This capability empowers machines to discern patterns and correlations within datasets, granting them valuable technical utility.^276–280^ It has rapidly evolved from being a concept in science fiction to an integral part of everyday life. It plays a fundamental role in various sectors, including self-driving cars,^281^ the energy sector,^282^ chemical science,^283^ web chats,^284^ spam filters,^285^ material science,^286^ and face recognition.^287^ ML is executed through artificial neural networks (ANNs) comprising interconnected computing elements known as artificial neurons. This terminology parallels the hypothesized functioning of biological neurons in living organisms. ANNs are primarily tasked with analyzing extensive sets of interrelated data inputs and determining an optimal set of weights that describe the correlations within the dataset. This capability proves especially beneficial when dealing with large datasets where discerning patterns or correlations may be challenging or unclear. The efficacy of an ANN model improves with the dataset's size and the refinement of the neural network's structure. Neurons within ANNs are organized into “layers” that connect the model's input and output. Each layer contains varying numbers of neurons, depending on the input and output characteristics. In the initial layer, each neuron receives input and transmits it to subsequent layer neurons by applying a weight. Identifying and optimizing these weights constitute the core of the computational process, often termed the “learning” process within ANNs. Commonly utilized ML models include the multilayer perceptron (MLP), support vector machine (SVM), decision tree (DT), relevance vector machine (RVM), long short-term memory (LSTM), and random forest (RF).

The integration of advanced nanosensors and machine learning has been shown to have promising applications in different industries and engineering systems, including human–machine interactions, the food industry,^288^ water quality monitoring,^289^ agriculture,^290^ healthcare, manufacturing processes, environmental monitoring, and smart homes. For instance, the integration between ML and nanosensors has been exploited in disease X detection.^291^ In this application, nanosensors are used to identify signals in biological samples from infected people triggered by one or more biomarkers. Using artificial intelligence-based cloud computing approaches, this integration helps handle huge data with high scalability, flexibility, and cost-effectiveness. The need for cloud computing is growing along with the development of nanosensors since these technologies can handle the processing, storing, and analysis of large amounts of data produced by sensors. As a result, cloud computing is vital to nanosensors, providing several benefits and influencing how they are used.^292^ The optical community has gradually incorporated ML and data science approaches into photonics research.^293^ This has resulted in several successful applications, such as optical microscopy and communication. Cloud ML in nanophotonics offers crucial computational resources for simulating complex optical structures, speeding up the creation of novel materials and devices, enabling optical simulations, design optimizations, and data processing, and facilitating the investigation of creative design approaches and technological advances.^294^ Furthermore, cloud computing gives wearable electronics access to more storage and computational power, accelerating the development of wearables that can process massive amounts of data in real time with high accuracy.

Additionally, cloud computing is the foundation for advanced Internet of Things (IoT) technologies, enabling computerized decision-making, intelligent home automation, and real-time data transmission.^295^ Cloud-based technologies provide remote management and control in robotics, allowing robots to access computational power beyond their onboard capabilities.^296^ Cloud computing is used by environmental monitoring systems to collect and process data from multiple sensors, resulting in more precise forecasts and prompt responses to environmental changes. Furthermore, ML-powered cloud healthcare platforms improve patient care and facilitate safe data exchange between healthcare professionals, leading to better health outcomes and easier access to medical research. In the era of quickly developing IoT technology, when massive amounts of data are regularly generated and analyzed, finding effective computing techniques has become crucial.

**Fig. 16:**
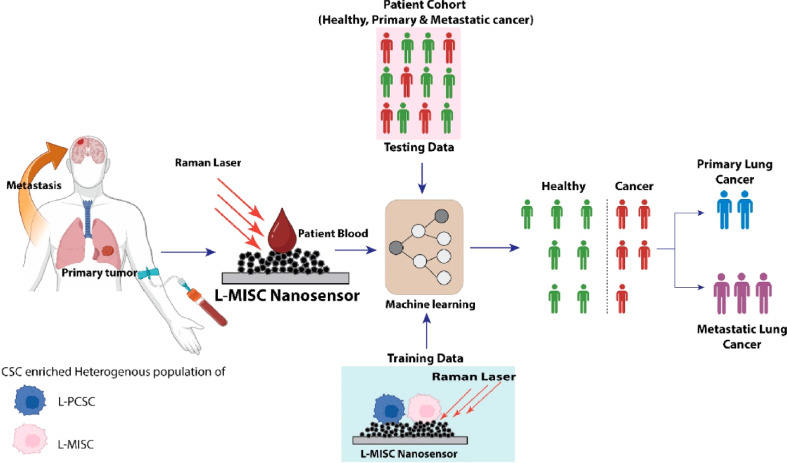
Lung cancer diagnosis using metastasis initiation stem cells nanosensor and ML.^298^

Medicine and healthcare have experienced rapid evolution and advancement, driven and shaped by the applications of data-driven, robust, and efficient ML technologies.^297^ These ML advancements improve diagnostics, treatment optimization, and overall healthcare management. Premachandran *et al.*^298^ identified metastatic signatures in the blood of lung cancer patients using a nanosensor designed for detecting lung cancer infections, as shown in Fig. 16. This nanosensor, which leveraged metastasis initiation stem cells, demonstrated the ability to detect lung cancer with just a tiny amount of blood—precisely, 5 μl. Tumorspheres generated from cancer cells, enriched with stem cells, served as the training data for the classification model. In contrast, lung fibroblast cells from a healthy cohort were used for training data. The classification model employed an ensemble of decision trees within a random forest to predict lung cancer. Test data were obtained from both cancer patients and healthy individuals. The machine learning model successfully classified the examined individuals with a specificity of 88% and a sensitivity of 100%. The developed machine learning algorithm effectively predicted the likelihood of lung cancer metastases. Achieving a sensitivity and specificity of 100%, this model enabled the prediction of metastasis, making it possible to diagnose both primary and metastatic lung cancer. The established model removes the necessity for time-consuming isolation procedures and presents a method for detecting biomarkers without labels. The nanosensors employed in this investigation provide significant improvements, enabling the precise identification of metastasis in the bloodstream with sensitivity at the level of individual cells. Yaari *et al.*^299^ investigated a perception-driven sensing system to detect various biomarkers in human biofluids. Using photoluminescence, they employed a sensor array based on DNA-linked single-walled carbon nanotubes. Using an artificial molecular perception system, a new approach was suggested for identifying multiple biomarkers in biofluids for disease diagnosis. Optical responses were harnessed to train machine learning models, including SVM, MLP, and RF, to identify gynecologic cancer biomarkers in laboratory-generated samples and patient fluids. Qureshi *et al.*^300^ emphasized the advantages of incorporating ML into biosensor design at an individual patient level, offering benefits for disease prediction and data interpretation considering the detection of SARS-CoV-2 disease as a case study. They employed a Bayesian optimizer to reverse the design of biosensors, employing pre-defined NMs, which enabled the creation of a programmable biosensor. This approach facilitates the attainment of desired chemical and optical characteristics with improved sensitivity. Kumar *et al.*^301^ introduced a highly sensitive gold/Ti_3_C_2_T_*x*_-coated-based sensor designed to diagnose cancer patients. A hybrid gold/Ti_3_C_2_T_*x*_ layer was applied circularly to induce surface plasmon resonance over the photonic crystal fiber. This coating method is based on the coupled mode theory. Simulations and numerical analyses were performed utilizing the finite element method. The resonance wavelength shift between cancerous and normal cell samples was measured to determine wavelength resolution and sensitivity.

Furthermore, they analyzed parametric variation optimization using an ML technique, achieving a good optimization accuracy with a little error of 0.01525. Sensitivity analysis with an MLP model exhibited a 2% error. The developed sensor, combined with the application of ML, indicates that this sensor has the potential to function as a rapid, efficient, and low-cost device for detecting infected cases.

In the field of materials science, discovering recipes that produce NMs with specific optical properties is a labor-intensive and time-consuming process. ML approaches could help discover novel NMs with varied compositions and rediscover NPs with diverse properties that rely on size and form. NPs must be created with carefully regulated properties for photovoltaics, chemical sensing, thermoelectrics, catalysis, medical diagnostics, or pharmaceutics.^302^ R. Mohammadzadeh kakhki and M. Mohammadpoor^303^ employed ML algorithms to optimize the synthesis process of carbon dots with enhanced properties, such as electrocatalytic activity, stability, and fluorescence. ML can effectively be used to screen carbon dot precursors and predict their properties. Mahata *et al.*^304^ optimized the synthesis process of SnO_2_ nanopetals used in nanosensor fabrication. Nayak *et al.*^305^ fabricated LiFe_5_O_8_-based nanosensors using a single-step hydrothermal technique. The sensor's selectivity issue was tackled using simpler ML algorithms, including K-nearest neighbor, RF, DT, and SVM. These algorithms are employed to enhance the sensor's ability to distinguish and selectively respond to specific target analytes. DT was declared the optimal classification model, emerging as the most effective with an impressive accuracy of 98%. This indicates the high performance and reliability of the developed ML model in accurately classifying and addressing the objectives of the task at hand.

Despite their high dimensionality, analytical chemistry has successfully applied ML tools to extract quantitative and qualitative data from complex measurements.^306^ The use of ML in analytical chemistry has proven valuable for enhancing the analysis and interpretation of intricate chemical data. Ge *et al.*^307^ developed a robust wireless intelligent approach for detecting mycophenolic acid in silage based on electrochemical nanosensor characteristics. The nanosensor electrode comprised zinc-cobalt metal–organic frameworks/titanium carbide and MXene/iron oxide-graphene oxide. The developed MLP model comprised one input, one output, and five neurons in the hidden layer. The developed ML model showed good computational fitting between pH as the input and mycophenolic acid as the output. Unfortunately, monitoring mycotoxins on a real-time basis had not been accomplished. Zhu *et al.*^308^ established an intelligent-based nanosensing platform by utilizing MLP, SVM, and RVM to ultra-trace carbendazim residues in rice and tea. The nanosensing materials were composed of Ag–Au nano shuttles/graphene-like titanium carbide. They were used for both surface-enhanced Raman scattering and electrochemical detection. RVM model outperformed MLP and SVM for intelligent analysis of carbendazim.

**Fig. 17:**
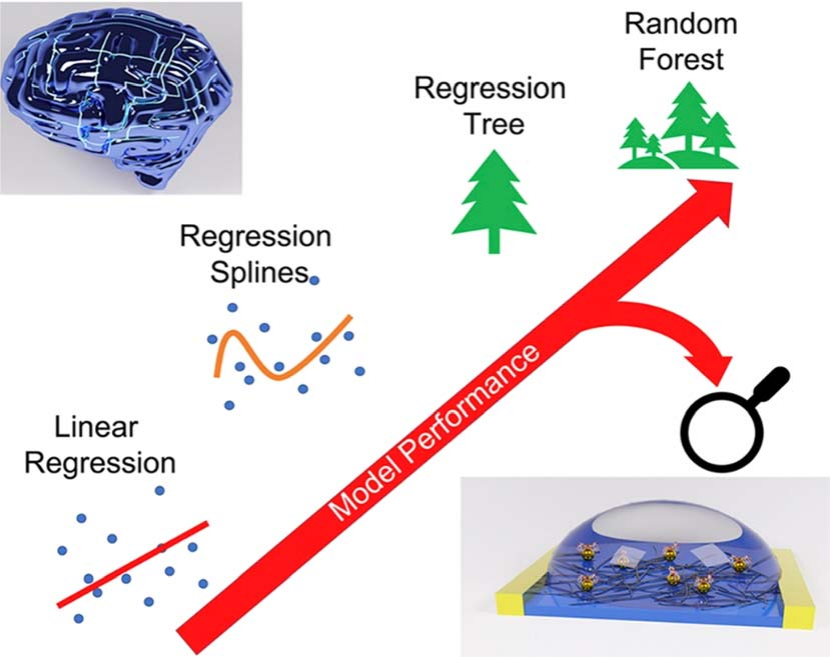
Electronic nanosensors calibration using linear regression, regression splines regression tree, and random forest.^309^

**Fig. 18:**
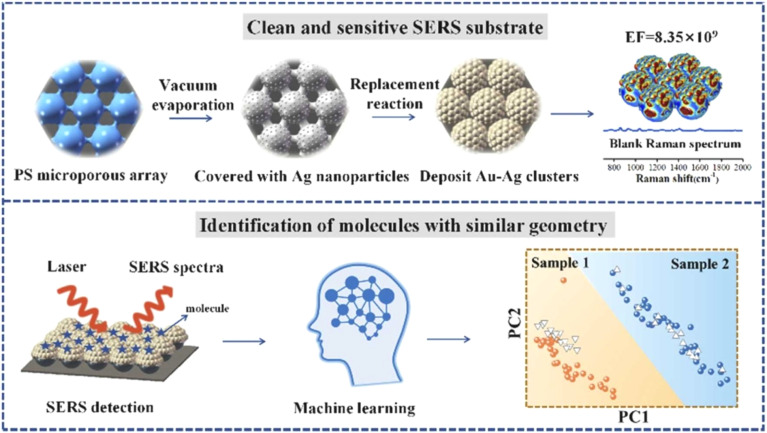
Detection of pollutants based on SERS sensors assisted by ML algorithms.^310^

Electronic sensing technology is already extensively employed to assess the quality of various products, contributing to enhancing product quality across diverse industries. These sensors are crucial in monitoring and controlling various parameters, ensuring products meet specified standards and quality criteria. The applications of electronic sensing sensors span multiple sectors, including manufacturing, agriculture, food production, and environmental monitoring, among others. ML methods have found extensive applications in solving real-world production problems, addressing challenges that are often difficult to tackle using traditional methods in various engineering fields. These methods have proven valuable in optimizing processes, predicting outcomes, identifying patterns, and enhancing decision-making across different engineering aspects, contributing to increased efficiency and improved solutions in complex scenarios. Bian *et al.*^309^ employed ML techniques to develop a calibration method for field-effect transistor devices based on carbon nanotubes, as shown in Fig. 17. The procedure commenced with the implementation of linear regression, which was then followed by the utilization of regression splines to accommodate the non-linearities present in the data. Regression trees were introduced to improve the model performance, and further enhancements were made by incorporating an RF model to reduce model variance. The resulting performance, assessed by *R*^2^, is estimated at 0.8260 using out-of-bag error. This method successfully prevents saturation and broadens the dynamic range of nanosensors by as much as 12 orders of magnitude in analyte concentrations. Additional exploration into the sensing mechanism involves examining the significance of features in each model tested. Employing machine learning methods to pinpoint features in the transistor signal that closely correlate with concentration shifts provides a valuable understanding of the carbon nanotube sensing mechanism. This contributes to the informed design of forthcoming nanosensors. Lin *et al.*^310^ introduced an ultra-sensitive and highly accurate method for identifying various pollutants, utilizing a cascade-enhanced nanosensor in conjunction with ML algorithms, as shown in Fig. 18. The surface-enhanced Raman scattering (SERS) substrate employed exhibits the capability to generate cascading electromagnetic energy, achieving a remarkable enhancement factor of up to 8.35 × 10^9^. This extraordinary enhancement is attributed to the synergistic combination of a micro-level polystyrene sphere porous array and nano-level Au–Ag clusters within the substrate. Leveraging its high cleanliness and ultra-sensitivity, the nanosensor successfully distinguished multiple hazardous pollutants with similar geometry and Raman peaks at ultra-low concentrations, supported by principal component analysis. The integration of this efficient and clean SERS substrate with artificial intelligence holds the potential to propel the application of SERS technology for the accurate identification of trace contaminants. The developed SERS substrate demonstrates significant potential and promising prospects in diverse fields, including food analysis, environmental monitoring, pharmaceuticals, and electrochemistry. An SVM model was constructed to discriminate between the Raman spectra of SERS fabricated through magnetron sputtering technology, achieving a correct classification percentage of 83%.^311^ Hu *et al.*^312^ demonstrated the efficacy of RF as a machine-learning model for predicting signals of SERS in a *trans*-1,2-bis(4-pyridyl)ethylene molecule situated on an Au substrate. The ML protocol accurately forecasts vibrational frequencies and Raman intensities using geometric descriptors derived from quantum chemistry simulations involving thousands of *ab initio* molecular dynamics conformations. The resulting spectra closely align with density functional theory calculations and experimental observations. Moreover, the protocol's robust transferability is evident in the predicted SERS responses of the molecule on diverse surfaces and under external influences such as electric fields and solvent environments. Wu *et al.*^313^ introduced an innovative method for developing high-performance SERS sensors by employing self-assembled Au NPs on glass capillaries. The surface self-assembly technique guarantees consistent and reproducible quality of SERS substrates, tackling the typical problem of poor reproducibility in traditional methods. The 30 nm Au nanoparticles chosen showcase excellent plasmonic properties and biocompatibility, rendering them well-suited for SERS applications. The analyzed substrate exhibits significant uniformity and repeatability, with a relative standard deviation of 12.1%. Machine learning methods like normalization, baseline correction, and smoothing were utilized in data processing to enhance the accuracy and reliability of the SERS analysis. Three separate clusters of spectral characteristics were distinguished using the K-means clustering algorithm. Furthermore, Principal Component Analysis (PCA) was employed to visually represent and interpret the clustering outcomes in a two-dimensional space, capturing roughly 86.74% of the data's variance. Lussier *et al.*^314^ reported the creation and utilization of ML algorithms combined with a SERS nanoprobe. This approach allowed for the simultaneous measurement of gradients for at least eight metabolites *in vitro* near various cell lines. The findings showed a significant increase in the secretion or consumption of lactate, glucose, ATP, glutamine, and urea within a 20 μm proximity to the cell surface compared to the bulk. The results also indicated that cancerous cells (HeLa) exhibited a higher glycolytic rate than fibroblasts (REF52), consistent with the expected phenotype. Both endothelial cells (HUVEC) and HeLa cells showed a notable increase in extracellular ATP compared to the control, highlighting the significance of extracellular ATP within the cancer's local environment. The ML-driven SERS optophysiology approach broadly applies to metabolites involved in cellular processes, establishing a versatile platform for studying cell biology. This technique holds potential applicability for addressing various significant challenges, including monitoring the effects of drug treatments on cells, viruses, and bacteria. Moreover, it may have relevance for *in vivo* experiments. Revignas and Amendola^315^ devised a straightforward attempt for detecting glutathione (GSH) utilizing surface Au NPs. The variation in the electric double layer of the Au NPs, influenced by increasing GSH concentration, leads to particle aggregation, resulting in a noticeable color change that can be measured. This phenomenon, commonly leveraged for optical sensing, has been incorporated into an undergraduate course to acquaint students with concepts related to NPs, colloids, colloidal stability, and sensor attributes such as selectivity, sensitivity, and detection range. They employed ML models to quantitatively correlate the corresponding absorption characteristics of Au-based sensors to analyte concentration.

Analyzing the toxicity of NPs is crucial for assessing the safety of NMs and understanding their potential impact on biological systems.^316^ However, traditional experimental methods for evaluating nanoparticle toxicity can be expensive and time-consuming. As an alternative approach, machine learning provides a solution for predicting cellular responses to NPs. This method leverages computational models and algorithms to analyze data and make predictions, offering a more efficient and cost-effective way to assess nanoparticle toxicity than traditional experimental methods. Ku *et al.*^317^ employed electrochemical sensors, semiconductor metal oxide sensors, and photoionization detection sensors to discern chemical hazards through the utilization of ML. This integrated sensor system, leveraging various sensing technologies, enables the identification and discrimination of different chemical substances. ML algorithms enhance the ability to analyze and interpret the sensor data effectively. Sensing data inputs were utilized in either image or numerical data, and the classification of chemical hazards with high accuracy was achieved in both cases. This flexibility in data input formats demonstrates the versatility and effectiveness of the approach, showcasing the ability to leverage diverse types of sensor data for accurate chemical hazard classification. The efficacy of ML-based gas discrimination is demonstrated by its ability to leverage even small amounts of gas sensing or purging data, typically input for around 30 seconds. This highlights the efficiency of the approach in extracting valuable information from relatively brief sensing periods, making it practical for real-time applications and enhancing the feasibility of gas discrimination tasks. Wang *et al.*^318^ developed a fluorescence sensor enhanced by ML for sulfide determination. This sensor incorporated a nanometal-organic framework (UiO-66-NH_2_) and protoporphyrin IX (Ppix). The blue fluorescence at 431 nm, originating from the UiO-66-NH_2_ moiety upon 365 nm excitation, served as an internal calibration reference signal. Simultaneously, the red fluorescence at 629 nm from the Ppix moiety served as the analytical signal, with intensity correlated to the amount of sulfides. The fluorescence color of the sensor gradually transitioned from blue to red with the sequential addition of copper and sulfide ions. This variation led to color characteristics based on RGB values corresponding to sulfide concentrations, enabling advanced data processing techniques through ML algorithms. They further developed an online data analysis model based on fluorescence image fingerprint extraction and ML algorithms to improve the precision and accuracy of sulfide determination. The model, employing Linear Discriminant Analysis (LDA) and undergoing rigorous cross-validation, demonstrated a significant positive relationship between red feature values and sulfide concentrations. This comprehensive approach presents a powerful tool for environmental monitoring and pollution detection. Long *et al.*^319^ developed an ML-assisted dual-channel visual sensor array to identify the origin of Lilium bulbs (BH). They utilized quantum dots and nanogold clusters as components in the sensor arrays. The presence of amino acids in Lilium bulbs induced an aggregation-induced fluorescence enhancement effect of nanogold clusters through hydrogen bonding. The reported outcomes revealed that the developed dual-channel visual sensor and the utilized ML model exhibited a high accuracy of 94.4% for identifying lilium bulbs from eight different origins. This achievement underscores the system's capability for rapid and accurate traceability of Lilium bulbs from various geographical locations. The success of this approach holds promising application prospects, particularly in the context of food traceability.

This review is anticipated to provide analytical chemists and researchers with a deeper understanding of the recent advancements in ML-assisted nanosensor arrays, particularly in various engineering applications. Considering the substantial advancements achieved by nanosensors in engineering fields, it is expected that machine learning-assisted nanosensor arrays based on functional NMs will find practical applications. However, integrating ML and nanosensors in real-life applications faces a significant obstacle—ultimately, a lack of trust in the model predictivity, primarily stemming from poor model interpretability. As datasets increase in dimensionality, researchers must resort to more intricate ML algorithms to enhance predictive performance. Nevertheless, these ML systems are frequently characterized as ‘black boxes' and face unclear and ambiguous decision-making issues, impacting their reliability.

Consequently, there has been a noticeable shift in emphasis towards ‘explainable machine learning’ aimed at enhancing model interpretability. This involves enabling algorithms to trace and elucidate their decision-making processes in a way researchers can understand. For instance, sophisticated techniques should be employed to assess the relative significance of input features in a constructed model. This information can offer better guidance for selecting scientifically relevant input features when interpreted alongside domain knowledge. Recently, advanced model frameworks have been developed to incorporate additional constraints, allowing the generation of various interpretable explanations that specifically include selected essential features. This becomes particularly valuable for generating more specific interpretations by employing different input feature subspaces. These subspaces can elucidate explanations for either a single or smaller sample subset. Incorporating explainable ML algorithms is crucial for instilling greater confidence in ML models' robustness and scientific validity. It facilitates their widespread integration into diverse environmental, healthcare, and agri-food settings. A trend will emerge involving integrating artificial intelligence, nanotechnology, and 3D-printed technology to develop innovative portable nanosensor arrays for exact applications. This review aims to promote rational design and construction of low-cost, rapid, selective, and sensitive nanosensors for various engineering applications.

## 8. Conclusion

This paper provides a comprehensive review of NMs-based nanosensors as well as the advancement that has occurred recently in the development of advanced materials and nanostructures, with their extraordinary combinations of thermal, electrical, mechanical, and optical properties, which has dramatically expanded the limits in the field of nanosensors. These advancements have led to a heightened interest in applications in various fields, such as environmental monitoring, the food industry, health monitoring, biomedical and industrial applications, and other safety and security controls.

The use of nanosensors has the potential to revolutionize multiple sectors by providing innovative solutions for detection, monitoring, and analysis. The healthcare field stands to benefit significantly from nanosensors, as they offer new possibilities for disease diagnostics, personalized medicine, and patient monitoring. Nanosensors empower individuals to take control of their health and well-being with their ability to detect biomarkers and monitor vital signs in real time.

In addition to healthcare, nanosensors also significantly impact other industries, such as electronics, manufacturing, aerospace, and defense. By enabling precise and real-time monitoring of critical parameters, nanosensors contribute to advancements in these sectors. These miniature sensors are smaller than human hair but offer high sensitivity and specificity in detecting various biomolecules and small-molecule metabolites.

Moreover, integrating machine learning techniques with nanosensor technologies opens up new opportunities for data exploration and predictions. Clustering, classification, and regression algorithms can analyze complex multidimensional datasets with high sample variability associated with nanosensor measurements. By combining these machine learning strategies with customizable nanosensor platforms, researchers can develop multifaceted strategies that further boost the performance of nanosensors.

Looking ahead, several vital areas emerge as pivotal for future research and development in the realm of nanosensors:

1- Material innovation: continued exploration and synthesis of novel nanomaterials (NMs) with enhanced properties and functionalities will be crucial. The research will focus on discovering materials that offer excellent stability, biocompatibility, and environmental sustainability.

2- Integration with emerging technologies: the convergence of nanosensors with cutting-edge technologies such as artificial intelligence, IoT, and blockchain could revolutionize data analysis, security, and sensor networking. Future work will likely explore how these integrations can lead to more intelligent, efficient, and interconnected sensing systems.

3- Enhanced fabrication techniques: developing more efficient, cost-effective, and scalable fabrication methods remains a priority. Future research should aim to streamline manufacturing processes while maintaining the high quality and performance of nanosensors.

4- Improving sensing abilities: efforts to enhance the sensitivity and selectivity of nanosensors for specific applications, particularly in the early detection of diseases or environmental pollutants, will be crucial.

## Conflicts of interest

There are no conflicts to declare.
